# Preventive and therapeutic effects of ginsenosides on myocardial ischemia-reperfusion injury in animal models: a systematic review and meta-analysis

**DOI:** 10.1186/s12872-026-05503-7

**Published:** 2026-01-14

**Authors:** Hongyi Yue, Yunfei Jia, Ruohao Sun, Zhuoyang Song, Wenhua Li

**Affiliations:** 1 Clinical Medical Research Center for Plateau Gastroenterological Disease of Xizang Autonomous Region, Xizang Minzu University, Shaanxi, 712082 China; 2 The Affiliated Hospital of Xizang Minzu University, Shaanxi, 712082 China; 3 Hainan Medical University, Haikou, 570100 China; 4 Department of Neurosurgery, Beijing Tiantan Hospital, Capital Medical University, Beijing, 100070 China

**Keywords:** Ginsenosides, Myocardial ischemia-reperfusion injury, Meta-analysis, Inflammation, Apoptosis, Oxidative stress

## Abstract

**Background:**

Myocardial ischemia-reperfusion injury (MIRI) markedly impairs cardiac functional recovery and represents a major determinant of adverse outcomes in patients with ischemic heart disease. Ginsenosides, the principal bioactive constituents of ginseng, exert significant cardioprotection against MIRI. This review systematically summarizes and analyzes in vivo (animal) studies to clarify the efficacy and underlying mechanisms of ginsenosides in MIRI.

**Methods:**

The PubMed, EMbase, Web of Science, Cochrane Library, CNKI, WanFang, and Cqvip databases were systematically searched from inception to 31 July 2024. In vivo studies evaluating ginsenosides pretreatment or post-treatment in models of MIRI were identified. Outcome measures comprised myocardial infarct size and indices of hemodynamic performance, myocardial injury, apoptosis, inflammation, and oxidative stress. A meta-analysis was conducted with RevMan 5.4 and Stata/MP 14.0.

**Results:**

Thirty-four eligible articles encompassing 505 experimental animals were included. Funnel plots, Egger’s tests, and sensitivity analyses confirmed the robustness of the findings. Compared with controls, ginsenosides treatment significantly reduced myocardial infarct size and improved hemodynamic indices (P < 0.0001). Ginsenosides also attenuated MIRI-induced elevations of lactate dehydrogenase, creatine kinase-MB, creatine kinase, malondialdehyde, tumor necrosis factor-α, interleukin-6, interleukin-1β, and cardiomyocyte apoptosis (P < 0.0001). Subgroup analysis further revealed that pre-ischemic ginsenosides administration conferred greater protection than post-reperfusion treatment.

**Conclusion:**

Ginsenosides play a significant role in the prevention and treatment of MIRI. Ginsenosides can reduce the area of myocardial infarction and improve myocardial damage through anti-inflammatory, antioxidative stress, anti-apoptosis, regulation of autophagy, and energy metabolism.

**Supplementary Information:**

The online version contains supplementary material available at 10.1186/s12872-026-05503-7.

## Introduction

Ischemic heart disease (IHD) is one of the most common and serious cardiovascular diseases in clinical practice. According to relevant epidemiological surveys, approximately 4 million people die from IHD in China each year [1]. Restoring blood perfusion is an effective and important method for the treatment of IHD, but the reperfusion process often leads to secondary injuries such as myocardial edema, cell membrane rupture, mitochondrial swelling, and excessive sarcomere contraction, which in turn leads to myocardial ischemia-reperfusion injury (MIRI) [2, 3]. MIRI severely affects survival after revascularization in patients with IHD and greatly reduces the benefit of reperfusion therapy and the recovery of cardiac function [4]. To date, the pathogenesis of MIRI has remained incompletely understood, and no effective therapeutic strategy is available. Consequently, identifying effective approaches to preventing and treating MIRI has become a major focus of cardiovascular research.

As a world-famous traditional Chinese medicinal herb, *Panax ginseng* C. A. Mey. (ginseng) has long been recognized for its significant therapeutic value; recent studies have increasingly indicated that its efficacy mainly relies on its primary active constituents, ginsenosides [5, 6]. Ginsenosides are triterpenoid glycosides divided into four types: protopanaxadiol, protopanaxatriol, ocotillol, and oleanolic acid. The types differ in structure, properties and activity, but their pharmacological effects are similar: antioxidant and anti-inflammatory actions, immune modulation and autophagy induction [7]. A substantial body of evidence indicates that ginsenosides confer marked cardioprotection in vivo and in vitro by suppressing homocysteine-induced superoxide-anion generation and endothelial dysfunction and by protecting human vascular endothelial cells from oxidized-LDL-mediated injury [8, 9]. In addition, ginsenoside pretreatment reverses the increases in reactive oxygen species production and the decreases in superoxide dismutase activity in vascular smooth muscle cells that are induced by resistin and acetylated LDL, thereby preventing vascular injury [10]. These pharmacological mechanisms align with the recognized therapeutic mechanisms of MIRI, making it highly significant to evaluate the cardioprotective effect of ginsenosides in MIRI treatment.

Thus far, although numerous preclinical studies have confirmed ginsenosides as promising agents against MIRI, their efficacy remains neither systematically summarized nor critically evaluated. To fill this gap, we provide a comprehensive assessment of the protective effects of ginsenosides on MIRI and delineate the underlying mechanisms, offering a theoretical framework for the prevention, treatment and clinical translation of MIRI.

## Methods

This article aims to reveal the effects of ginsenosides on MIRI in preclinical studies through systematic review and meta-analysis. In this study, we strictly adhered to the guidelines of the PRISMA statement [11]. Furthermore, to ensure transparency and traceability, this meta-analysis was registered with PROSPERO (registration number: CRD42024569208).

### Search strategy

A systematic literature search was conducted across seven databases: PubMed, Embase, Web of Science, Cochrane Library, CNKI, WanFang and Cqvip. We aimed to identify all potentially relevant studies to ensure the meta-analysis was based on the most comprehensive and up-to-date scientific evidence. The search period extended from the inception of the database to July 31, 2024. The search formula was constructed as follows: [(Ginseng) OR (Ginsenosides) OR (Ginseng Saponin) OR (Panaxosides) OR (Sanchinosides)] AND [(Myocardial Ischemia) OR (Ischemic Heart Disease) OR (Myocardial Reperfusion) OR (Myocardial Ischemia-Reperfusion Injury) OR (Injuries, Myocardial Reperfusion) OR (Myocardial Reperfusion Injuries) OR (Reperfusion Injuries, Myocardial) OR (Reperfusion Injury, Myocardial) OR (Injury, Myocardial Reperfusion)]. To minimize the risk of missing relevant studies, we also tracked the citations of selected articles.

### Inclusion and exclusion guidelines

According to the PICOS principle, literature was included in this meta-analysis if the following conditions were met: (1) population: animals; (2) intervention: administration of ginsenosides drugs (either before or after MIRI); (3) comparison: the control group was given saline or no treatment for the experimental animals; (4) outcome: myocardial infarction size, apoptosis rate, hemodynamic indices [maximum rate of left ventricular pressure rise (+ dp/dtmax), maximum rate of left ventricular pressure decrease (-dp/dtmax), left ventricular ejection fraction (LVEF), left ventricular systolic pressure (LVSP), left ventricular end-diastolic pressure (LVEDP)], myocardial injury markers [lactate dehydrogenase (LDH), creatine kinase (CK), creatine kinase MB (CK-MB)], oxidative stress markers [superoxide dismutase (SOD), malondialdehyde (MDA)], and inflammation markers [tumor necrosis factor-alpha (TNF-α), interleukin-6 (IL-6), and interleukin-1β (IL-1β)]; (5) study design: animal studies of MIRI were constructed using ligation of the left anterior descending (LAD) branch of the coronary artery or other methods (langendorff perfusion system; ligation of the right atrial vein).

The literature will be excluded if the following conditions are met: (1) during administration, ginsenosides are administered along with other drugs; (2) low relevance to the core themes of this study; (3) reviews, abstracts, conferences, clinical trials, and in vitro experiments; (4) the study suffers from missing data; (5) the MIRI modeling was unsuccessful.

### Data extraction

After applying the predefined inclusion and exclusion criteria, we screened the articles and then independently extracted the data. This information includes: (1) the name of the first author and the year in which the article was published; (2) species, sex, weekly age, and weight of the animals used in the study; (3) types of narcotic drugs; (4) methods of constructing disease models and the duration of ischemia-reperfusion; (5) duration of the intervention group and method of intervention; (6) classes of ginsenosides monomers; (7) outcome indicators and the mechanism of action of the treatment. When the intervention arm included different ginsenoside doses, we combined the subgroup data into a single treatment group using the methodology recommended by the Cochrane Handbook [12]. This approach ensures that the combined data accurately reflects the treatment effects of the different conditions. In addition, when articles presented data graphically, we digitized them with GetData Graph Digitizer 2.22 for statistical analysis [13].

### Risk of bias and quality assessment

Included articles were independently assessed based on the SYRCLE statement’s recommended risk assessment scale for experiments on animals [14]. The assessment process covered the following aspects: (A) the level of detail at which the sequence was generated; (B) whether animal characteristics were accurately described at baseline; (C) whether the allocation sequence is adequately concealed; (D) whether the animal’s rearing process is randomized; (E) blinding of researchers; (F) randomness in outcome measurement; (G) blinding researchers during outcome assessment; (H) treatment of Incomplete Results; (I) whether there is a risk of selective reporting of results; (J) whether other potential biases exist. All entries were categorized as having a high, low, or unclear risk of bias, and the summary was visualized in RevMan 5.4. The reliability of outcomes was then assessed using GRADE [15]. Correction and verification of the raw data were conducted by the corresponding author, WH. In cases of disagreement during the evaluation process, the final decision was also made by WH.

### Statistical analysis

The forest plot was generated using RevMan 5.4 (The Cochrane Collaboration, London, England), and the statistical significance level was set at *P* < 0.05. In forest plots, parameter variables were consistently represented as mean ± standard deviation. For studies that reported data as medians and quartiles, the means and standard deviations were estimated using the methods outlined by Wan et al. and Hozo et al. [16, 17]. For the effect indicators in the forest plots, the standardized mean difference (SMD) was consistently used due to variations in age and sex among the included studies, as well as the involvement of multiple species [18]. In order to assess whether there was publication bias in the included studies and whether the results were stable, we utilized Stata/MP 14.0 (StataCorp, College Station, TX, USA) software and employed a variety of methods for testing. Specifically, this involved Egger’s test, funnel plot, and sensitivity analysis [19, 20]. When publication bias was detected in the results, we employed the trim-and-fill method to account for potentially missing studies and recalculated the effect size. Additionally, to assess heterogeneity among the included studies, we performed the *I²* test. If *P* < 0.10 or *I²* > 50%, this indicated significant heterogeneity between studies, in which case we selected the random-effects model for analysis. If *P* > 0.10 or *I²* < 50%, heterogeneity was not significant, and we chose the fixed-effects model for analysis [21]. In addition, subgroup analyses, meta-regression analyses, and sensitivity analyses were conducted to investigate potential causes of heterogeneity. The subgroup and meta-regression analyses focused on five outcomes: myocardial infarct size, LDH, CK-MB, SOD, and MDA, as these outcomes were covered in a sufficient number of studies. Sensitivity analysis involved removing each study individually and observing the impact of this action on *I²* values.

## Results

### Selection of included studies

**Fig. 1:**
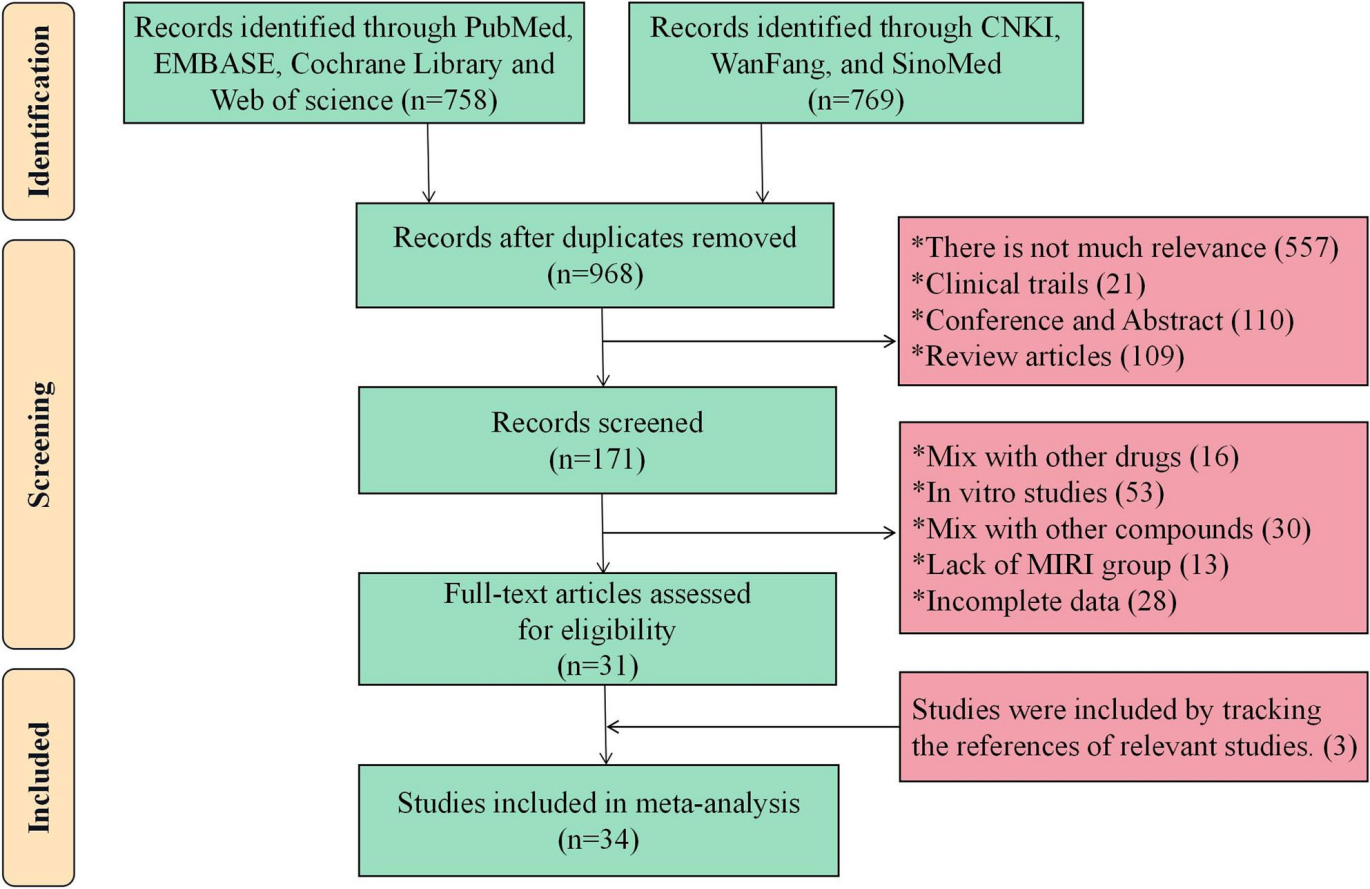
Flowchart of study selection based on PRISMA

The process of literature retrieval is shown in Fig. 1. After implementing the established search strategy, we screened a total of 1,527 articles in seven databases, including 758 articles in English and 769 articles in Chinese. During the initial screening process, 559 duplicate studies were identified and removed using NoteExpress software, leaving 968 candidates. For further screening, the abstracts and titles of the remaining articles were briefly read by two researchers, HY and YJ. Among these, 557 articles with low relevance to the core topic, 110 conference abstracts, 21 clinical studies, and 109 reviews were excluded. A detailed reading of the remaining 171 articles led to the further exclusion of 140 papers. Among these, 46 were mixed with other drugs or compounds, 53 were in vitro studies, 13 lacked model groups, and 28 had incomplete data. We then tracked down three eligible studies from the citations of Zheng et al. [22] Ultimately, 34 papers were included in this meta-analysis [23–56].

### Characteristics of the included studies

This study pooled 34 studies covering 505 animals, with 253 animals in the intervention group and 252 animals in the model group. Of all the studies, SD rats were the most common, appearing in 24 studies [25–28, 30–33, 35–37, 40–49, 54–56]. This was followed by Wistar rats, which were used in 6 studies [24, 38, 39, 50, 51, 53]. Additionally, one study utilized Duncan-Hartley guinea pigs [23], and two studies involved C57 BL/6 J mice [29, 34]. The MIRI model is commonly constructed using coronary artery ligation. The duration of ischemia was 0.5 h in most studies, 0.75 h in two studies [42, 53], and 1 h in one study [23]. Regarding the time to reperfusion, a reperfusion time of 2 h was more common, while other studies varied from 1 to 24 h. Ether and pentobarbital were the primary anesthetics utilized during model construction. Nevertheless, it is noteworthy that seven studies did not specify the use of anesthetics [29, 31, 33, 36, 45, 51, 53]. The main modes of administration of ginsenosides include intragastric administration, intravenous injections, and intraperitoneal injections. Regarding the use of drug monomers, 17 studies used ginsenoside Rb [24, 25, 29, 31, 32, 35–38, 41, 42, 45, 47–50, 55], 8 studies used ginsenoside Rg [28, 30, 33, 34, 39, 44, 52, 56], 4 studies used ginsenoside Rh [26, 27, 40, 46], and 5 studies used other types of ginsenoside monomers [23, 43, 51, 53, 54]. Other basic characteristics are shown in Table 1.

**Table 1:** Basic characteristics of the included studies

Study and Year	Species and gender	Week old and Weight	Anesthetic	Modeling method	Treatment group		MI/RI Time	Drug (Monomer)	Pretreatment	Outcomes	Mechanism of action
					Time of administration	Mode of administration					
Aravinthan et al. 2015	Duncan-Hartley guinea pigs, Male	ND, 300–350 g	Pentobarbital	Ligation of the right atrial vein	14 days	Take orally	1 h/2 h	Ginseng total saponin	No	b, c, e, g, h, k, n, o	Inflammation↓, Oxidative stress↓
Chen 2012	Wistar rats, Male	ND, 220–260 g	Ethylether	Ligation of LAD	7 days	Intragastric administration	0.5 h/3 h	Ginsenoside Rb1	No	j, k	Antioxidant enzyme activity↑, Oxidative stress↓, eNOS level↑
Cui et al. 2017	SD rats, Male	ND, 230–270 g	Urethane	Ligation of LAD	30 min before MIRI	Intravenous injection	0.5 h/1.5 h	Ginsenoside Rb1	Yes	a, b, c, e, f	RhoA signaling pathway↓, Energy metabolism disorder↓, Apoptosis↓
Fan et al. 2021	SD rats, Male	ND, 160–200 g	Pentobarbital	Ligation of LAD	10 days	Intravenous injection	0.5 h/4 h	Ginsenoside Rh2	No	a, j, o	NLRP3 protein↓, Inflammation↓, Oxidative stress↓
Fan et al. 2023	SD rats, Male	1–8 weeks, ND	Pentobarbital	Ligation of LAD	10 days before MIRI	Intravenous injection	0.5 h/24 h	Ginsenoside Rh2	Yes	a, d, g, h, i, j, k	Nrf2↑/HO-1↑/NLRP3↓, Inflammation↓, Oxidative stress↓
He et al. 2015	SD rats, Female or Male	ND, 250–280 g	Urethane	Ligation of LAD	15 days before MIRI	Intragastric administration	0.5 h/2 h	Ginsenoside Rg1	Yes	a, l, m, o	NF-κB signaling pathway↓, Inflammation↓
Jiang et al. 2021	C57BL/6 mice, Male	6–8 or 8–12 weeks, ND	ND	Ligation of LAD	10 min before MIRI	ND	0.5 h/15min, 24 h, 14d, 28d	Ginsenoside Rb1	Yes	a, d	NADH dehydrogenase activity↓
Li et al. (1) 2020	SD rats, ND	ND	Chloral hydrate	Ligation of LAD	At the time of ligation	Intramyocardial injection	0.5 h/2 h	Ginsenoside Rg3	No	a, g, h, i, j, k	Inflammation↓, Oxidative stress↓, Fibrosis↓
Li et al. (2) 2020	SD rats, Male	ND, 200–300 g	ND	Langendorff perfusion system	30 min before MIRI	ND	0.5 h/0.5 h,1 h	Ginsenoside Rb1	Yes	a, b, c	Autophagy↓, Myocardial contractility↑
Li et al. 2016	SD rats, Male	ND, 200–250 g	Pentobarbital	Ligation of LAD	5 min before reperfusion	Intravenous injection	0.5 h/2 h	Ginsenoside Rb1	No	a, m	p38α MAPK phosphorylation↓, Apoptosis↓
Li et al. 2018	SD rats, Male	ND, 240–260 g	ND	Ligation of LAD	150 min before MIRI	Femoral venous catheter infusion	0.5 h/1.5 h	Ginsenoside Rg1	Yes	a, b, c, e, f	RhoA ↓/ ROCK↓, Activity of mitochondria respiratory Complexes↑, ATP5D↑
Li et al. 2022	C57BL/6 mice, Male	12 weeks, 22–25 g	Pentobarbital	Ligation of LAD	5 min before reperfusion	via caudal tail injection	0.5 h/4 h	Ginsenoside Rg2	No	a, d, m	TAK1 phosphorylation↑, Necroptosis↓
Liu et al. 2012	SD rats, Male	ND, 250–300 g	Pentobarbital	Ligation of LAD	10 min before MIRI	ND	0.5 h/2 h	Ginsenoside Rb1	Yes	a, l	PI3K↑/Akt↑, Apoptosis↓
Liu et al. 2014	SD rats, Male	ND, 200–220 g	ND	Ligation of LAD	3 days	Take orally	0.5 h/2 h	Ginsenoside Rb3	No	a, g, h, j, k, m, n	Inflammation↓, Oxidative stress↓, Apoptosis↓
Liu et al. 2020	SD rats, Male	7–8 weeks, 220–240 g	Isoflurane	Ligation of LAD	3 days	Take orally	0.5 h/2 h	Ginsenosides Rb2 and Rb3	No	a, b, c, e, f, g, h, j, k, l, m, n	Inflammation↓, Oxidative stress↓, Apoptosis↓
Qu et al. 2007	Wistar rats, Female or Male	ND, 240–260 g	Ethylether	Ligation of LAD	7 days	Intragastric administration	0.5 h/24 h	Ginsenoside Rb	No	g, h, j, k	Antioxidant enzyme activity↑, Oxidative stress↓, Platelet aggregation activity↓
Rao et al. 2013	Wistar rats, Male	ND, 200–230 g	Ethylether	Ligation of LAD	7 days	Intragastric administration	0.5 h/24 h	Ginsenoside Rg2	No	j, k	Oxidative stress↓, eNOS level↑
Shao et al. 2018	SD rats, Male	ND, 180–250 g	Pentobarbital	Ligation of LAD	30 min before ischemia	Intraperitoneal injection	0.5 h/24 h	Ginsenoside Rh2	Yes	j, k, n	Inflammation↓, Oxidative stress↓, Peripheral blood EPCs↑
Shi et al. 2011	SD rats, ND	ND, 230–260 g	Chloralose	Ligation of LAD	3 days	Take orally	0.5 h/24 h	Ginsenoside Rb3	No	a, g, h, j, k	Oxidative stress↓, Microcirculatory disturbance↓
Wang et al. 2008	SD rats, Male	ND, 260–320 g	Pentobarbital	Ligation of LAD	10 min before ischemia	Intravenous injection	0.75 h/2 h	Ginsenoside Rb1	Yes	a, g, h, i	PI3K↑ /P-Akt↑
Wang et al. 2013	SD rats, Male	ND, 270–320 g	Pentobarbital	Ligation of LAD	30 min before MIRI	Intraperitoneal injection	0.5 h/3 h	Ginsenoside Rd	Yes	a, b, c, e, f, g, i	ROS↓, Apoptosis↓
Wang et al. 2015	SD rats, Male	ND, 260–280 g	Pentobarbital	Ligation of LAD	30 min before MIRI	Intravenous injection	0.5 h/3 h	Ginsenoside Rg3	Yes	a, b, c, e, f, g, i, j	Bcl-2↑/Bax↓, P-Akt↓/P-eNOS↓, Apoptosis↓
Wang et al. 2016	SD rats, Male	ND, 180–240 g	ND	Ligation of LAD	5 min before reperfusion	Intravenous injection	0.5 h/2 h	Ginsenoside Rb1	No	a, m	p38α phosphorylation↓, Apoptosis↓, Inflammation↓
Wang et al. 2017	SD rats, Male	ND, 180–200 g	Chloral hydrate	Ligation of LAD	7 days before MIRI	Intraperitoneal injection	0.5 h/2 h	Ginsenoside Rh3	Yes	a, g, i, j	Bcl-2↑/Bax↓, Oxidative stress↓
Wu et al. 2011	SD rats, Male	ND, 250–300 g	Pentobarbital	Ligation of LAD	20 min before MIRI	Intravenous injection	0.5 h/2 h	Ginsenoside Rb1	Yes	a, g, h, l	Cardiomyocyte apoptosis↓, Cardiac dysfunction↓
Wu et al. 2016	SD rats, Female or Male	ND, 200–250 g	Pentobarbital	Ligation of LAD	2 days	Intragastric administration	0.5 h/2 h	Ginsenoside Rb3	No	a, g, h, j, k	anti-lipid peroxide activities↑, Oxidative stress↓, Inflammation↓
Xia et al. 2011	SD rats, Male	ND, 220–280 g	Pentobarbital	Ligation of LAD	10 min before MIRI	Intravenous injection	0.5 h/2 h	Ginsenoside Rb1	Yes	a, g, i, j, k	Expression of eNOS↑, Oxidative stress↓
Xue et al. 2020	Wistar rats, Female	ND, 250–280 g	Pentobarbital	Ligation of LAD	3 days before MIRI	Intragastric administration	0.5 h/6 h	Ginsenoside Rb2	Yes	a, d, g, h, j, k, m, n, o	SIRT1↑, Oxidative stress↓, Inflammation↓
Xue et al. 2023	Wistar rats, Male	ND, 210–230 g	ND	Ligation of LAD	7 days before MIRI	Intragastric administration	0.5 h/6 h	Ginsenoside Rc	Yes	a, d, g, h, l	Mitochondrial oxidative stress and apoptosis↓, SIRT1↑
Xu et al. 2016	Rats, Male	ND, 200–230 g	Ethylether	Ligation of LAD	5 days	Intraperitoneal injection	ND	Ginsenoside Rg2	No	a, j, k	Antioxidant activity↑, Free radical oxidative damage↓
Ye et al. 2023	Wistar–Kyoto rats, Female	8 weeks, ND	ND	Ligation of LAD	5 days	Intragastric administration	0.75 h/ND	Ginsenoside Re	No	a, d, g, h	miR-144-3p↑/SLC7A11↑
Zeng et al. 2015	SD rats, Male	ND, 270–320 g	Pentobarbital	Ligation of LAD	30 min before reperfusion	Intraperitoneal injection	0.5 h/2 h	Ginsenoside Rd	Yes	a, b, c, d, f, g, i	Nrf2↑/HO-1↑, Cardiac dysfunction↓
Zhang et al. 2010	SD rats, Male	ND, 220–280 g	Pentobarbital	Ligation of LAD	10 min before ischemia	Intravenous injection	0.5 h/2 h	Ginsenoside Rb1	Yes	a, g, i, j, k	Oxidative stress↓, Free radical oxidative damage↓
Zhang et al. 2016	SD rats, Male	ND, 270–290 g	Chloralose	Ligation of LAD	7 days	Intragastric administration	0.5 h/24 h	Ginsenoside Rg3	No	b, c, d, e, f, l, m, o	Apoptosis↓, Inflammation↓

*ND* No description, *MIRI* Myocardial ischemia/reperfusion injury, *h* Hour, *LAD* Left anterior descending branch, *SD* Sprague-Dawley, *eNOS* Endothelial nitric oxide synthase, *Nrf2* NFE2-related factor 2, *HO-1* Heme oxygenase-1, *NLRP3* NOD-like receptor protein 3, *NADH* Nicotinamide adenine dinucleotide, *TAK1* TGF-beta activated kinase 1, *ROS* Reactive oxygen species, *SIRT1* Silent information regulator 1, *↓* reduction vs baseline, *↑* increase vs baseline

^a^myocardial infarction size, ^b^maximum rate of left ventricular pressure rise, ^c^maximum rate of left ventricular pressure decrease, ^d^left ventricular ejection fraction, ^e^left ventricular systolic pressure, ^f^left ventricular end-diastolic pressure, ^g^lactate dehydrogenase, ^h^creatine kinase MB, ^i^creatine kinase, ^j^superoxide dismutase, ^k^malondialdehyde, ^l^apoptotic rate, ^m^tumor necrosis factor-alpha, ^n^interleukin-6, ^o^interleukin-1β

### Quality assessment of included studies

**Fig. 2:**
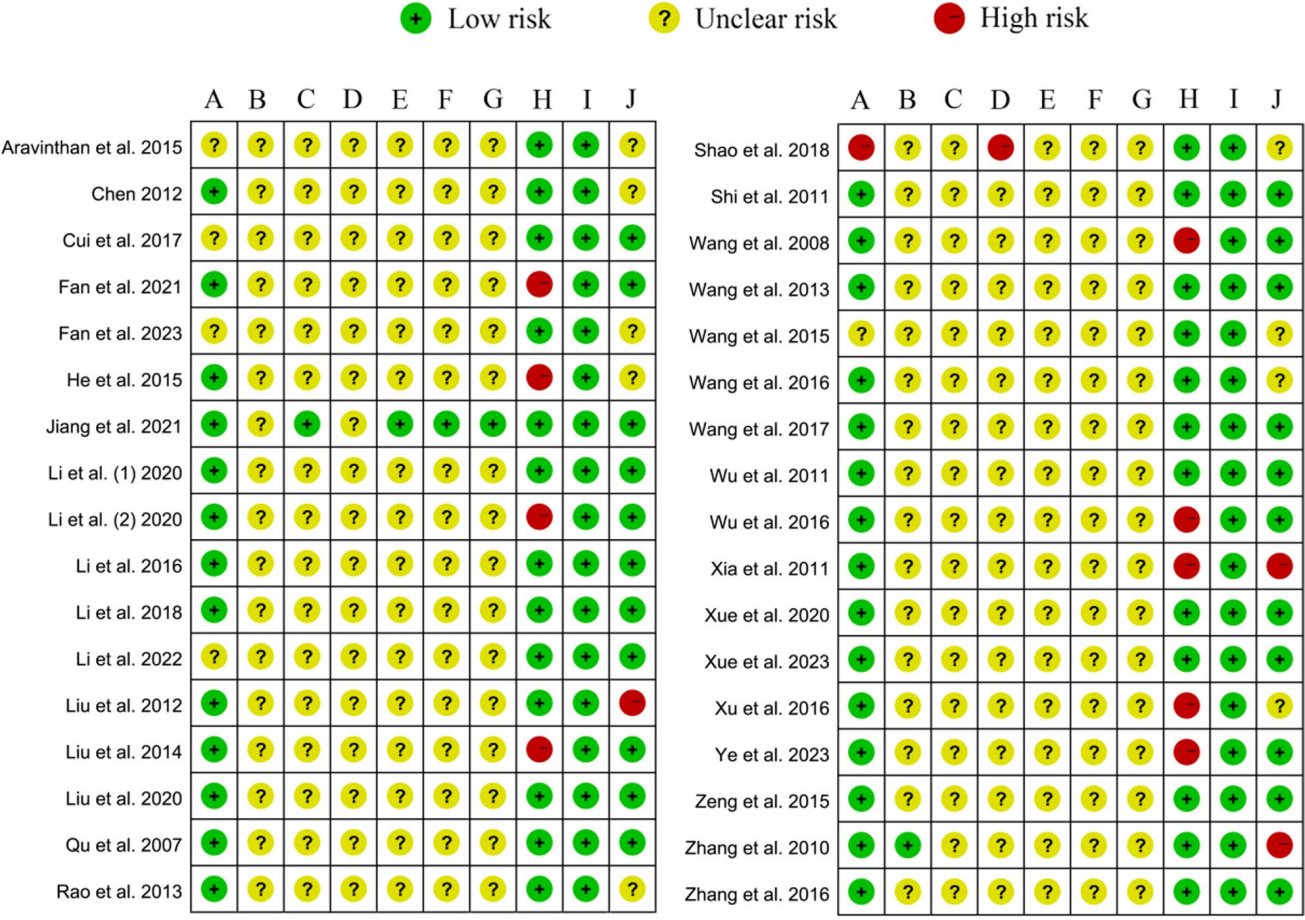
Risk of bias map of included studies based on the SYRCLE tool. **A**: Generation of random sequences; **B**: Characteristics of the baseline; **C**: Assign hidden situations; **D**: Randomness of animal feeding; **E**: Blinding of the experimenter; **F**: Randomness of outcome assessments; **G**: Blinding of outcome assessments; **H**: Integrity of result data; **I**: Selective reporting of results; **J**: Other biases

We assessed the quality of the included studies using SYRCLE, a risk of bias assessment tool for animal experiments, and the relevant results were displayed in Fig. 2. Sequence generation and animal feeding were randomized in most of the studies. Only one study was rated as high risk because the sequence generation was grouped according to body weight, and some animals were on a high-fat diet during feeding. Furthermore, only one study explicitly reported randomization of animal baseline characteristics, animal grouping, investigator blinding, outcome measure blinding, and outcome assessment. The remaining studies were rated as an unclear risk because these aspects were not adequately described. None of the studies selectively reported results. Nine studies were rated as high risk in terms of animal loss because they experienced animal loss [26, 28, 31, 36, 42, 48, 49, 52, 53]. Regarding other risks of bias, four studies were also rated as high risk because of possible errors in the unit of analysis [35, 47, 49, 55].

### Quality evaluation of outcome indicators

This study provided a comprehensive assessment of all outcome indicators based on the GRADE rating methodology. The results showed (Table 2) that the proportion of outcome indicators of high quality was 33.33% (5/15), that of low quality was 53.33% (8/15), and that of moderate and very low quality were each 6.67% (1/15). The risk of bias and publication bias were the main reasons for downgrading the quality of evidence. In addition, due to exaggerated data for some of the outcome indicators, we had to adjust the quality of these results downward accordingly.

**Table 2:** A GRADE summary of the protective effect of ginsenosides on MIRI

Outcomes	Relative effect (95% CI)	Limitations	Inconsistency	Indirectness	Imprecision	Publication bias	Quality
Infarct size	SMD, 3.09 (2.49, 3.69)	−	+	+	+	−	⨁⨁◯◯, Low
+dp/dtmax	SMD, 2.73 (1.69, 3.77)	+	+	+	+	+	⨁⨁⨁⨁, High
-dp/dtmax	SMD, 2.11 (1.42, 2.80)	+	+	+	+	+	⨁⨁⨁⨁, High
LVEF	SMD, 3.11 (1.89, 4.33)	+	+	+	+	+	⨁⨁⨁⨁, High
LVSP	SMD, 2.63 (1.63, 3.62)	+	+	+	+	+	⨁⨁⨁⨁, High
LVEDP	SMD, 3.88 (2.10, 5.66)	+	+	+	+	+	⨁⨁⨁⨁, High
LDH	SMD, 3.05 (2.25, 3.86)	−	+	+	+	−	⨁⨁◯◯, Low
CK-MB	SMD, 3.09 (2.03, 4.15)	−	+	+	+	−	⨁⨁◯◯, Low
CK	SMD, 6.32 (4.09, 8.54)	−	+	+	+	−	⨁⨁◯◯, Low
SOD	SMD, 2.30 (1.65, 2.94)	−	+	+	+	−	⨁⨁◯◯, Low
MDA	SMD, 1.97 (1.24, 2.70)	−	−	+	+	+	⨁⨁◯◯, Low
Apoptotic rate	SMD, 2.79 (1.33, 4.26)	+	+	−	+	+	⨁⨁⨁◯, Moderate
TNF-α	SMD, 4.43 (2.41, 6.45)	−	+	+	+	−	⨁⨁◯◯, Low
IL-6	SMD, 2.41 (1.09, 3.73)	−	+	−	+	−	⨁◯◯◯, Critically low
IL-1β	SMD, 2.63 (1.97, 3.29)	−	+	−	+	+	⨁⨁◯◯, Low

*LVEF* Left ventricular ejection fraction, *LVSP* Left ventricular systolic pressure, *LVEDP* Left ventricular end-diastolic pressure, *LDH* Lactate dehydrogenase, *CK-MB* Creatine kinase MB, *CK* Creatine kinase, *SOD* Superoxide dismutase, *MDA* Malondialdehyde, *TNF-*α Tumor necrosis factor-alpha, *IL-6* Interleukin-6, *IL-*1β Interleukin-1β, *SMD* Standardized mean difference, *WMD* Weighted mean difference, ⨁⨁⨁⨁ high certainty, ⨁⨁⨁◯ moderate certainty, ⨁⨁◯◯ low certainty, ⨁◯◯◯ very low certainty

^−^downgrade, ^+^not downgrade, ^+^dp/dtmax, Maximum rate of left ventricular pressure rise; ^-^dp/dtmax, Maximum rate of left ventricular pressure decrease

### Meta-analysis results

#### Effect of ginsenosides on myocardial infarction size

**Fig. 3:**
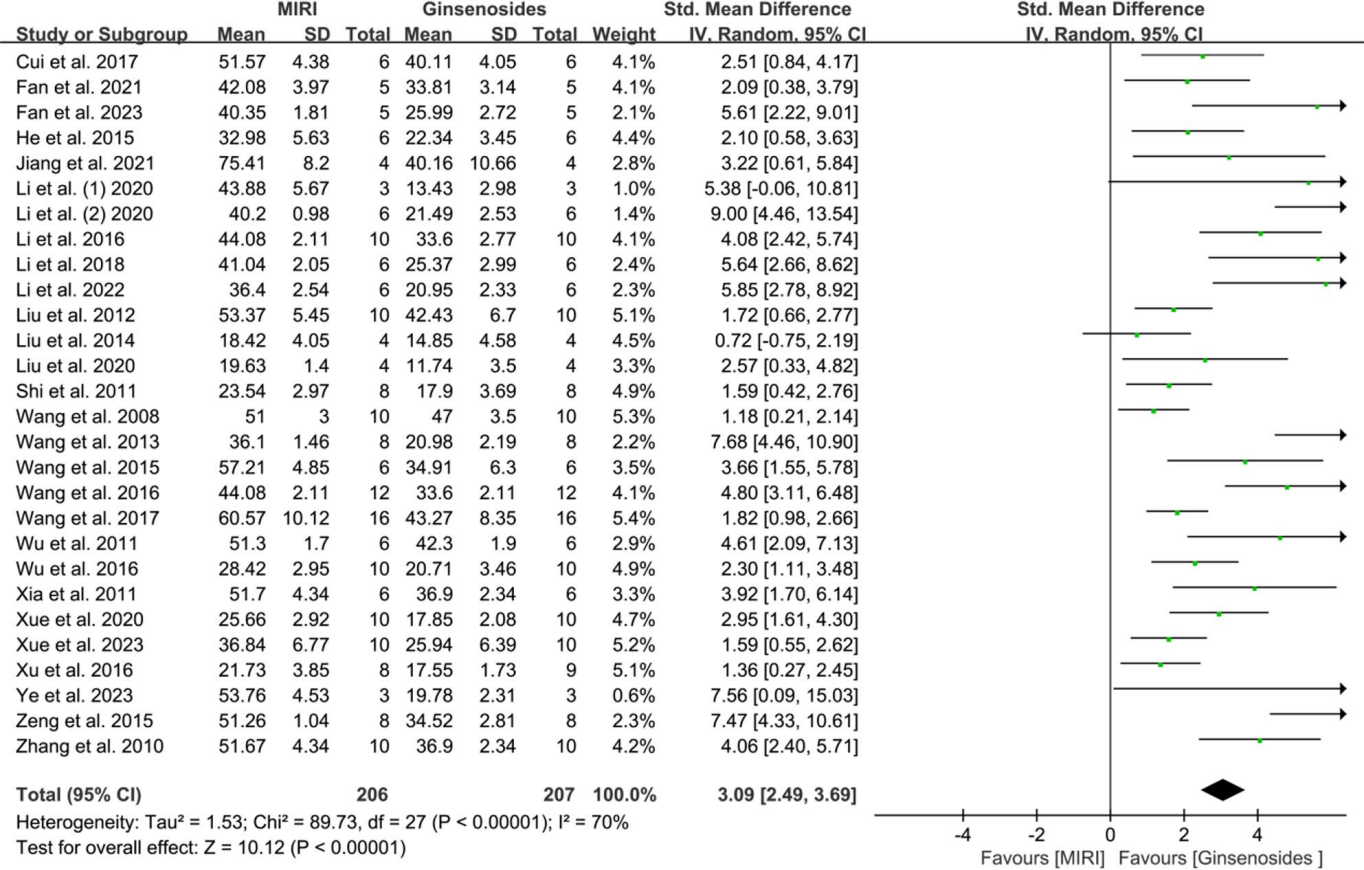
Forest plot of the effect of ginsenosides on the size of myocardial infarction

Measurement of myocardial infarction size (IS) was reported in 28 included studies [25–37, 41–55], involving 206 animals in the control group and 207 animals in the administration group. Because of significant heterogeneity in the determination of myocardial infarct size, the results were pooled and analyzed using a random-effects model (*I*^*2*^ = 70.00%, *P <* 0.00001). The results are shown in Fig. 3, where the use of ginsenosides significantly reduced the size of myocardial infarction (SMD = 3.09, 95% CI = 2.49–3.69, *P* < 0.00001). In addition, considering the significant heterogeneity in this analysis, we conducted a sensitivity analysis to investigate the reasons for this (Supplementary Fig. 1). After excluding each study individually, the total effect sizes and *I*^*2*^ values remained unchanged, indicating the robustness of our results.

#### Effects of ginsenosides on post-MI cardiac function

In order to verify the beneficial effects of ginsenosides on cardiac function, we examined five cardiac function indexes: +dp/dtmax, -dp/dtmax, LVEF, LVSP, and LVEDP. The results of the heterogeneity test showed + dp/dtmax *I*^*2*^ = 77.00%, -dp/dtmax *I*^*2*^ = 57.00%, LVEF *I*^*2*^ = 69.00%, LVSP *I*^*2*^ = 69.00%, LVEDP *I*^*2*^ = 87.00%. Consequently, all outcome indicators were analyzed using a random-effects model in a pooled manner. In addition, sensitivity analyses of the five outcome indicators showed that our results were reliable and stable (Supplementary Fig. 2).

**Fig. 4:**
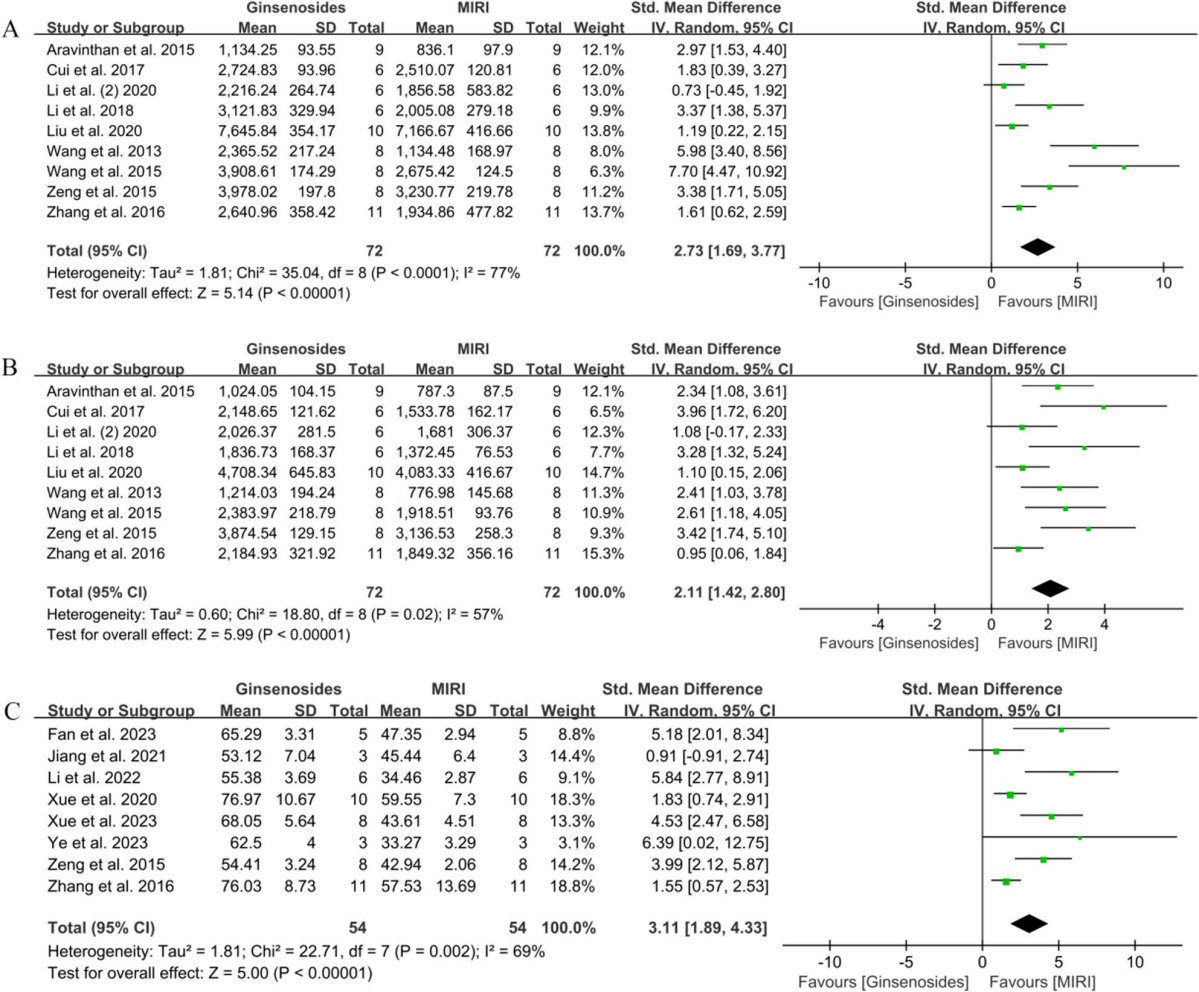
Forest diagram of the effect of ginsenosides on cardiac function. **A** + dp/dtmax, **B** **-**dp/dtmax, **C** LVEF

**Fig. 5:**
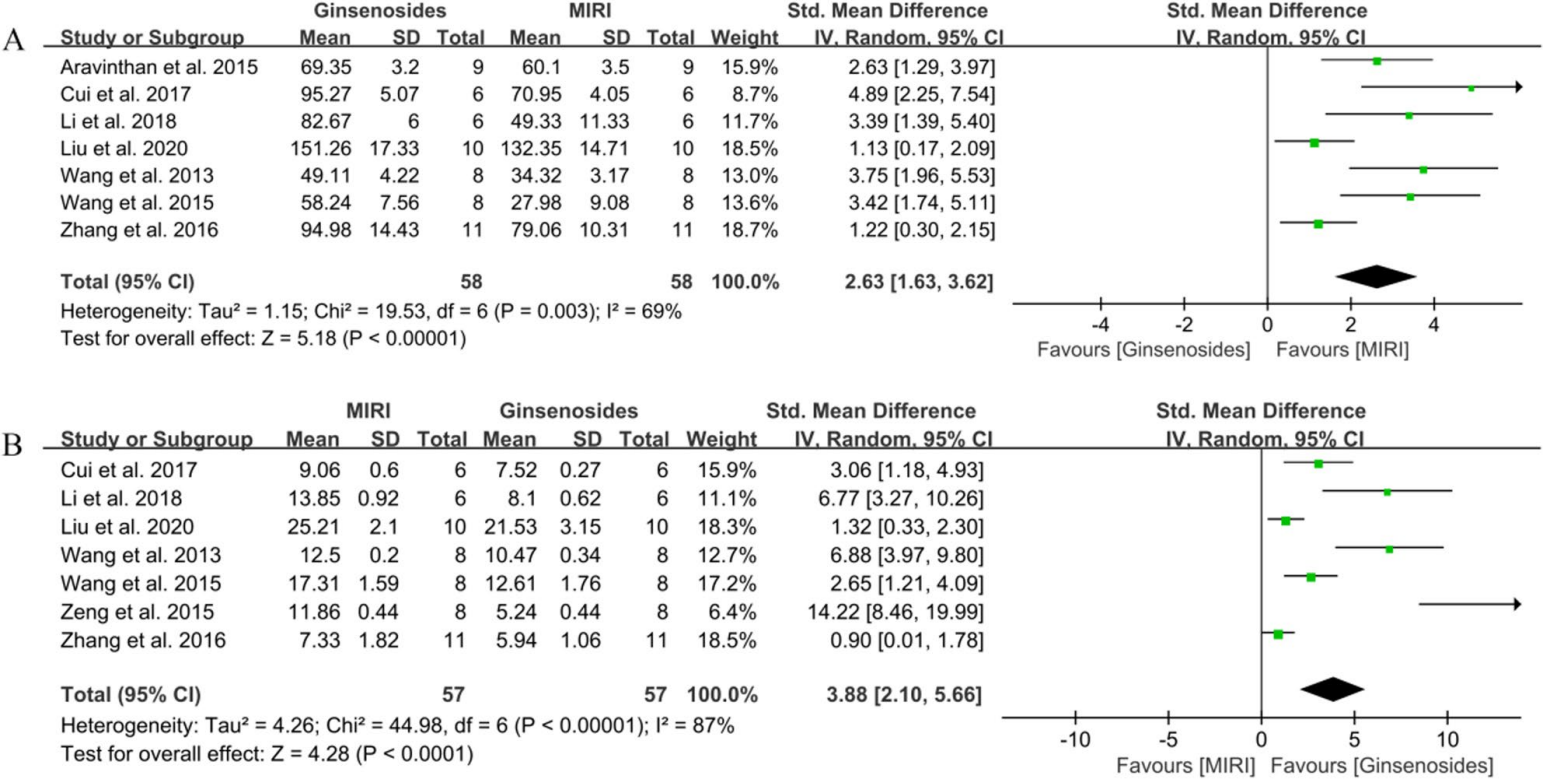
Forest diagram of the effect of ginsenosides on cardiac function. **A** LVSP, **B** LVEDP

First, there are nine articles that reported the therapeutic effects of ginsenosides on + dp/dtmax and -dp/dtmax [23, 25, 31, 33, 37, 43, 44, 54, 56]. These articles included a total of 72 animals in the control group and 72 animals in the administration group, as depicted in Fig. 4A and B. The use of ginsenosides significantly reversed the decrease in control + dp/dtmax (SMD = 2.73, 95% CI = 1.69–3.77, *P* < 0.00001). Simultaneously, the decrease in -dp/dtmax was also reversed (SMD = 2.11, 95% CI = 1.42–2.80, *P* < 0.00001). Secondly, eight studies reported the effect of ginsenosides on LVEF [27, 29, 34, 50, 51, 53, 54, 56], with 54 animals in the control group and 54 animals in the administered group. The results are shown in Fig. 4C, where LVEF was significantly higher in the administered group compared to the control group (SMD = 3.11, 95% CI = 1.89–4.33, *P* < 0.00001). The effect on LVSP was then determined in seven studies involving 58 animals in the dosing group and 58 animals in the control group [23, 25, 33, 37, 43, 44, 56]. As shown in Fig. 5A, ginsenosides were able to cause a significant increase in LVSP (SMD = 2.63, 95% CI = 1.63–3.62, *P* < 0.00001). Finally, seven studies (57 animals each in control and administered groups) reported the effect of ginsenosides on LVEDP [25, 33, 37, 43, 44, 54, 56]. Treatment with ginsenosides significantly reduced LVEDP (SMD = 3.88, 95% CI = 2.10–5.66, *P* < 0.0001). Specific results are shown in Fig. 5B.

#### Effect of ginsenosides on myocardial damage

Considering that the results of the heterogeneity test showed significant heterogeneity (LDH: *I*^*2*^ = 81.00%, CK-MB: *I*^*2*^ = 83.00%, CK: *I*^*2*^ = 89.00%), three markers of myocardial injury were evaluated using a random-effects model to explore the potential benefits of ginsenosides on myocardial injury. The results of the sensitivity analysis showed that the findings were robust for all three markers (Supplementary Fig. 3).

**Fig. 6:**
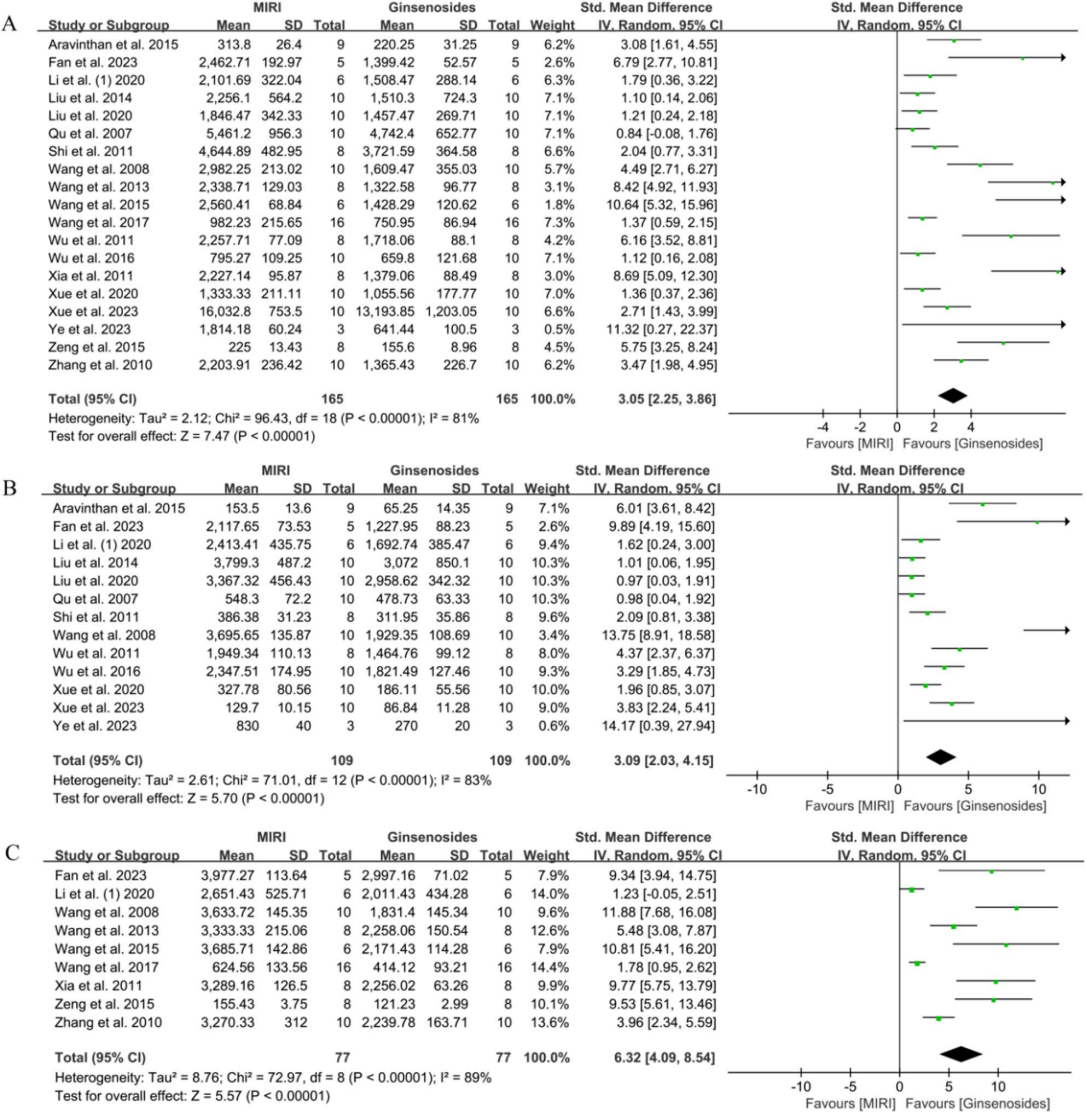
Forest plot of the effect of ginsenosides on markers associated with myocardial injury. **A** LDH, **B** CK-MB, **C** CK

A pooled analysis of 19 animal studies (165 animals each in control and administered groups) showed that ginsenosides significantly down-regulated LDH secretion compared to controls (SMD = 3.05, 95% CI = 2.25–3.86, *P* < 0.00001, Fig. 6A) [23, 27, 30, 36–38, 41–44, 46–51, 53–55]. Not only that, ginsenosides also reduced serum levels of CK-MB [23, 27, 30, 36–38, 41, 42, 47, 48, 50, 51, 53] (*n* = 318, SMD = 3.09, 95% CI = 2.03–4.15, *P* < 0.00001, Fig. 6B) as well as CK [27, 30, 42–44, 46, 49, 54, 55] (*n* = 154, SMD = 6.32, 95% CI = 4.09–8.54, *P* < 0.00001, Fig. 6C).

#### Effects of ginsenosides on apoptosis and oxidative stress

**Fig. 7:**
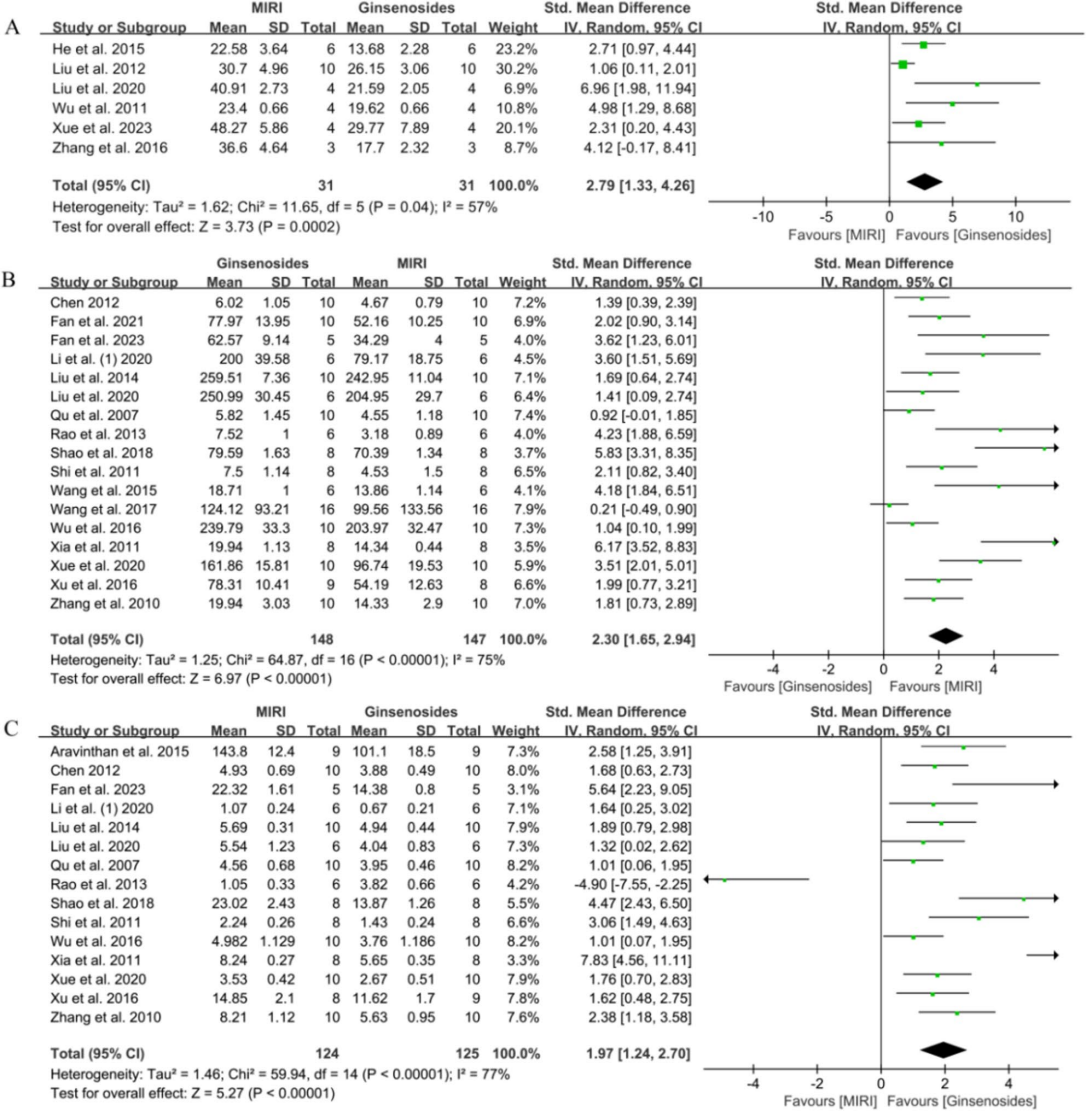
Forest plot of the effect of ginsenosides on indicators related to apoptosis and oxidative stress. **A** Cardiomyocyte apoptosis rate, **B** SOD, **C** MDA

The effect of ginsenosides on cardiomyocyte apoptosis was reported in six studies (31 animals each in control and administered groups) [28, 35, 37, 47, 51, 56]. The results of the pooled analysis are shown in Fig. 7A, where ginsenosides effectively resisted apoptosis in cardiomyocytes (SMD = 2.79, 95% CI = 1.33–4.26, *P* = 0.0002 < 0.001, heterogeneity: *I*^*2*^ = 57.00%). Sensitivity analysis showed this result to be stable (Supplementary Fig. 4).

To assess the effect of ginsenosides on oxidative stress, we analyzed the expression of SOD and MDA. Seventeen of these studies measured SOD [24, 26, 27, 30, 36–41, 44, 46, 48–50, 52, 55], and it was evident that there was a significant increase in the level of SOD in the treatment group (SMD = 2.30, 95% CI = 1.65–2.94, *P* < 0.00001, heterogeneity: *I*^*2*^ = 75.00%, Fig. 7B). Regarding the measurement of MDA, a pooled analysis of 15 studies [23, 24, 27, 30, 36–41, 48–50, 52, 55] showed that ginsenoside administration reduced MDA expression (SMD = 1.97, 95% CI = 1.24–2.70, *P* < 0.00001, heterogeneity: *I*^*2*^ = 77.00%, Fig. 7C).

#### Effect of ginsenosides on myocardial inflammation

Ginsenosides had good anti-inflammatory properties and effectively protected the myocardium from inflammatory responses. In this pooled analysis, we mainly observed the expression levels of three inflammatory factors: IL-1β, IL-6, and TNF-α. Due to the significant heterogeneity of IL-6 and TNF-α, analyses were performed using a random-effects model (IL-6: *I*^*2*^ = 81.00%, TNF-α: *I*^*2*^ = 90.00%). The heterogeneity of IL-1β metrics was not significant, so analyses were conducted using a fixed-effects model (IL-1β: *I*^*2*^ = 37.00%). Sensitivity analyses showed that all outcome indicators were stable (Supplementary Fig. 5).

**Fig. 8:**
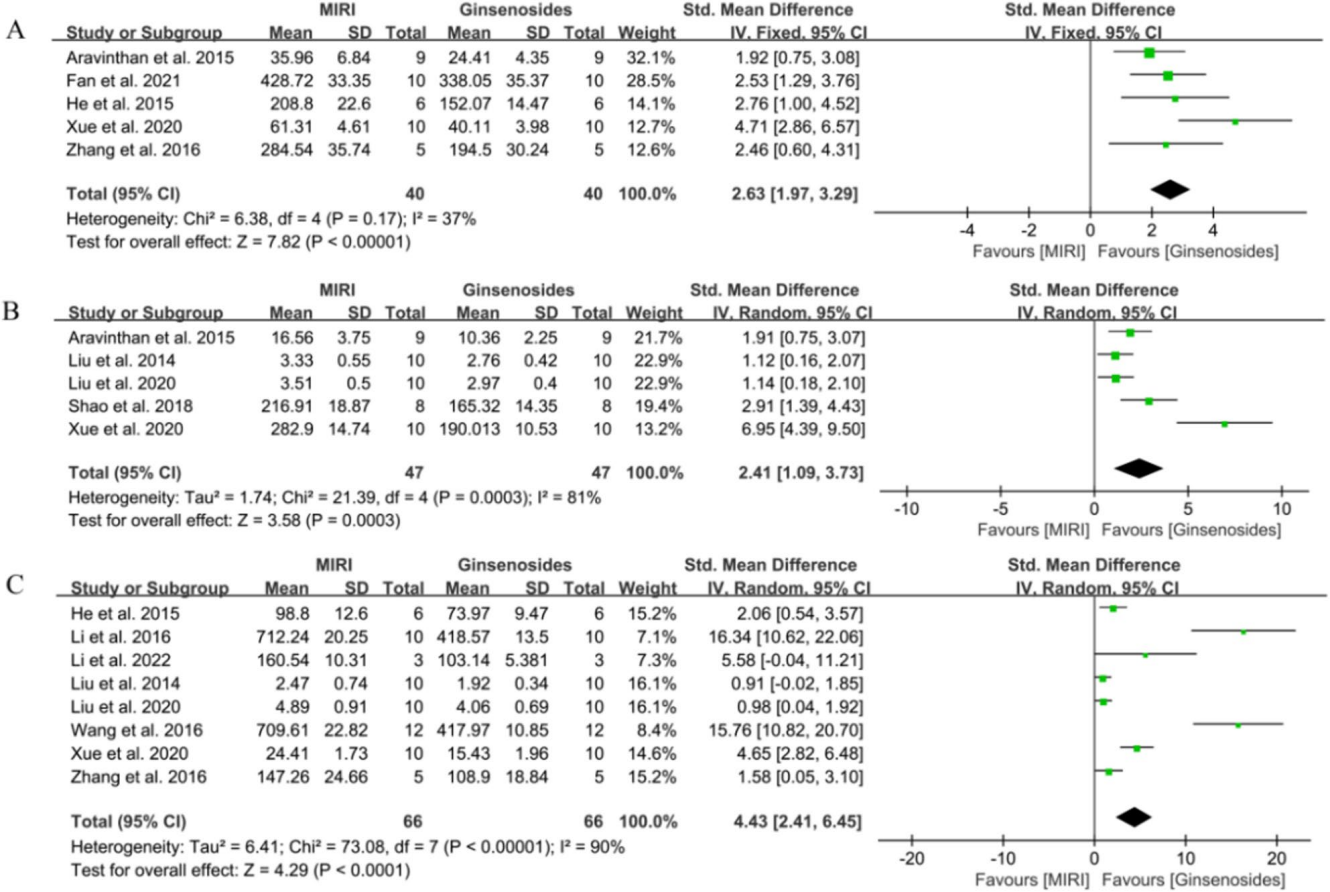
Forest plot of the effect of ginsenosides on myocardial inflammation-related indicators. **A** IL-1β, **B** IL-6, **C** TNF-α

In a pooled analysis of five studies [23, 26, 28, 50, 56] (40 animals per group), we observed a statistically significant reduction in IL-1β in the ginsenosides-treated versus control group (SMD = 2.63, 95% CI = 1.97–3.29, *P* < 0.00001, Fig. 8A). Additionally, the use of ginsenosides significantly reduced IL-6 [23, 36, 37, 40, 50] (SMD = 2.41, 95% CI = 1.09–3.73, *P =* 0.0003 < 0.01, Fig. 8B) and TNF-α [28, 32, 34, 36, 37, 45, 50, 56] (SMD = 4.43, 95% CI = 2.41–6.45, *P* < 0.0001, Fig. 8C).

### Results of subgroup analysis

First, subgroup analyses were performed on predefined five outcome indicators according to the type of ginsenosides (Table 3A). Subgroup analyses of IS showed that ginsenoside Rb (SMD = 2.85, 95% CI = 2.11–3.58, *P* < 0.01), ginsenoside Rg (SMD = 3.43, 95% CI = 1.85–5.01, *P* < 0.01), ginsenoside Rh (SMD = 2.47, 95% CI = 0.97–3.96, *P* < 0.01), and Other (SMD = 5.73, 95% CI = 1.51–9.94, *P* < 0.01) subgroups still significantly reduced myocardial infarct size, and the heterogeneity remained significant (Ginsenoside Rb: *I*^*2*^ = 68.00%; Ginsenoside Rg: *I*^*2*^ = 68.00%; Ginsenoside Rh: *I*^*2*^ = 56.00%; Other: *I*^*2*^ = 88.00%). Subgroup analyses of LDH showed that only the ginsenoside Rb (SMD = 2.43, 95% CI = 1.50–3.36, *P* < 0.01, *I*^*2*^ = 81.00%) and Other (SMD = 4.70, 95% CI = 2.69–6.72, *P* < 0.01, *I*^*2*^ = 72.00%) subgroups were able to observe benefits, and the heterogeneity remained significant for both. Subgroup analysis of the three outcome indicators, CK-MB, SOD, and MDA, showed that the protective effect of ginsenosides persisted. However, in a subgroup analysis of MDA outcome metrics, we found that the protective effect of the ginsenoside Rg subgroup on MDA was no longer present (SMD = -0.27, 95% CI = -3.24–2.69, *P* = 0.857, *I*^*2*^ = 91.00%). Subgroup difference tests indicated that different ginsenosides did not produce significant differences in the outcome measures of IS (*P* = 0.48, *I*^*2*^ = 0.00%), LDH (*P* = 0.21, *I*^*2*^ = 34.50%), and SOD (*P* = 0.16, *I*^*2*^ = 45.4%).

**Table 3:** Subgroup analyses based on (A) ginsenoside class, (B) pretreatment, and (C) species

Subgroups Outcomes (A)	Ginsenosides Rb				Ginsenosides Rg				Ginsenosides Rh				Other				Subgroups Differences
	SMD	95% CI	*P*	I^2^	SMD	95% CI	*P*	I^2^	SMD	95% CI	*P*	I^2^	SMD	95% CI	*P*	I^2^	
IS	2.85	[2.11, 3.58]	< 0.01	68%	3.43	[1.85, 5.01]	< 0.01	68%	2.47	[0.97, 3.96]	< 0.01	56%	5.73	[1.51, 9.94]	< 0.01	88%	*P* = 0.48, *I*^*2*^ = 0.0%
LDH	2.43	[1.50, 3.36]	< 0.01	81%	5.83	[−2.81, 14.47]	0.186	90%	3.71	[−1.55, 8.97]	0.167	85%	4.70	[2.69, 6.72]	< 0.01	72%	*P* = 0.21, *I*^*2*^ = 34.5%
CK-MB	2.46	[1.34, 3.58]	< 0.01	84%	1.62	[0.24, 3.00]	0.022	-	9.89	[4.19, 15.60]	< 0.01	-	5.06	[2.61, 7.51]	< 0.01	51%	*P* = 0.007, *I*^*2*^ = 75.4%
SOD	1.87	[1.21, 2.53]	< 0.01	64%	3.20	[1.97, 4.43]	< 0.01	39%	2.64	[0.55, 4.73]	0.013	89%	-	-	-	-	*P* = 0.16, *I*^*2*^ = 45.4%
MDA	1.92	[1.24, 2.59]	< 0.01	64%	−0.27	[−3.24, 2.69]	0.857	91%	4.78	[3.03, 6.52]	< 0.01	0%	2.58	[1.25, 3.91]	< 0.01	-	*P* = 0.007, *I*^*2*^ = 75.0%

**Subgroups** **Outcomes (B)**	**Pretreatment**								**No pretreatment**								**Subgroups** **Differences**
	**SMD**		**95% CI**		***P***		***I*** ^***2***^		**SMD**		**95% CI**		***P***		***I*** ^***2***^		
IS	3.37		[2.56, 4.17]		< 0.01		73%		2.72		[1.78, 3.66]		< 0.01		67%		*P* = 0.3, *I*^*2*^ = 5.0%
LDH	4.69		[3.26, 6.12]		< 0.01		85%		1.49		[0.93, 2.04]		< 0.01		40%		*P* < 0.0001, *I*^*2*^ = 94.0%
CK-MB	5.65		[2.94, 8.35]		< 0.01		87%		2.04		[1.09, 3.00]		< 0.01		74%		*P* = 0.01, *I*^*2*^ = 83.5%
SOD	3.40		[1.68, 5.11]		< 0.01		88%		1.71		[1.24, 2.18]		< 0.01		33%		*P* = 0.06, *I*^*2*^ = 71.2%
MDA	3.89		[2.12, 5.65]		< 0.01		78%		1.38		[0.64, 2.11]		< 0.01		72%		*P* = 0.01, *I*^*2*^ = 85.0%

**Subgroups** **Outcomes (C)**	**Rats**						**Mice**						**Duncan-Hartley guinea pigs**				**Subgroups** **Differences**
	**SMD**		**95% CI**		***P*** **and** ***I***^***2***^		**SMD**		**95% CI**		***P*** **and** ***I***^***2***^		**SMD (95% CI)**		***P*** **and** ***I***^***2***^		
IS	3.01		[2.40, 3.62]		*P* < 0.01, *I*^*2*^ = 70.0%		4.41		[1.85, 6.97]		*P* = 0.20, *I*^*2*^ = 39.0%		-		-		*P* = 0.30, *I*^*2*^ = 7.60%
LDH	3.07		[2.23, 3.91]		*P* < 0.01, *I*^*2*^ = 82.0%		-		-		-		3.08 [1.61, 4.55]		*P* < 0.01		*P* = 0.99, *I*^*2*^ = 0.0%
CK-MB	2.79		[1.76, 3.83]		*P* < 0.01, *I*^*2*^ = 82.0%		-		-		-		6.01 [3.61, 8.42]		*P* < 0.01		*P* = 0.02, *I*^*2*^ = 82.8%
MDA	1.92		[1.15, 2.70]		*P* < 0.01, *I*^*2*^ = 78.0%		-		-		-		2.58 [1.25, 3.91]		*P* < 0.01		*P* = 0.40, *I*^*2*^ = 0.0%

*IS* Infarct size, *LDH* Lactate dehydrogenase, *CK-MB* Creatine kinase MB, *SOD* Superoxide dismutase, *MDA* Malondialdehyde

Secondly, to thoroughly investigate the preventive and therapeutic effects of ginsenosides on MIRI, we conducted subgroup analyses based on whether ginsenosides were administered as pretreatment or not (Table 3B). Pretreatment subgroup analyses showed that ginsenosides were beneficial for all five outcome measures: IS (SMD = 3.37, 95% CI = 2.56–4.17, *P* < 0.01, *I*^*2*^ = 73.00%), LDH (SMD = 4.69, 95% CI = 3.26–6.12, *P* < 0.01, *I*^*2*^ = 85.00%), CK-MB (SMD = 5.65, 95% CI = 2.94–8.35, *P* < 0.01, *I*^*2*^ = 87.00%), SOD (SMD = 3.40, 95% CI = 1.68–5.11, *P* < 0.01, *I*^*2*^ = 88.00%), and MDA (SMD = 3.89, 95% CI = 2.12–5.65, *P* < 0.01, *I*^*2*^ = 78.00%), and the heterogeneity was significant for all of them. Not only that, the protective effect of ginsenosides persisted in the subgroup without pretreatment. It should be noted that the heterogeneity of the protective effect of ginsenosides on LDH and SOD was not significant in this subgroup (LDH: *I*^*2*^ = 40.00%, SOD: *I*^*2*^ = 33.00%). Overall, differences among subgroups were observed regarding the interventions [LDH: (*P <* 0.05, *I*^*2*^ = 94.00%); CK-MB (*P* = 0.01, *I*^*2*^ = 83.50%); SOD (*P* = 0.06, *I*^*2*^ = 71.20%); MDA: (*P* = 0.01, *I*^*2*^ = 85.50%)].

Finally, we conducted subgroup analyses based on species (Table 3C). The results showed that in rats, ginsenosides had statistically significant effects favoring the intervention on IS (SMD = 3.01, 95% CI = 2.40–3.62, *P* < 0.01, *I*^*2*^ = 70.00%), LDH (SMD = 3.07, 95% CI = 2.23–3.91, *P* < 0.01, *I*^*2*^ = 82.00%), CK-MB (SMD = 2.79, 95% CI = 1.76–3.83, *P* < 0.01, *I*^*2*^ = 82.00%), and MDA (SMD = 1.92, 95% CI = 1.15–2.70, *P* < 0.01, *I*^*2*^ = 78.00%), although all exhibited considerable heterogeneity. In mice, no significant therapeutic effect of ginsenosides on alleviating IS was observed, with low heterogeneity (SMD = 4.41, 95% CI = 1.85–6.97, *P >* 0.05, *I*^*2*^ = 39.00%). However, due to insufficient data, the effects of ginsenosides on LDH, CK-MB, and MDA in mice and Duncan-Hartley guinea pigs remain unclear.

### Meta-regression analysis

To fully investigate sources of heterogeneity, a meta-regression analysis was conducted for difficult-to-cluster confounding factors, including anesthetic type, time of administration, mode of administration, duration of ischemia, and duration of reperfusion. The results are shown in Table 4. We found that these confounding factors were not sources of heterogeneity for the outcome measures IS, LDH, SOD, and MDA. However, time of administration (*P* = 0.009 < 0.05) and duration of ischemia (*P* = 0.006 < 0.05) were identified as sources of heterogeneity for the CK-MB outcome measure.

**Table 4:** Meta-regression analysis was performed with anesthetic type, time of administration, mode of administration, duration of ischemia, and duration of reperfusion as covariates

Outcomes	Heterogeneity Factor	Coefficient	Std. Err	t	*P* Value	95% CI
IS	Anesthetic type	−0.2109764	0.1713932	−1.23	0.231	[−0.5664241, 0.1444713]
	Time of administration	0.1195327	0.0918508	1.30	0.207	[−0.0709542, 0.3100195]
	Mode of administration	−0.1070075	0.2231552	−0.48	0.636	[−0.569803, 0.355788]
	Duration of ischemia	−0.7215826	0.8845747	−0.82	0.423	[−2.556078, 1.112913]
	Duration of reperfusion	−0.2578801	0.2436062	−1.06	0.301	[−0.7630884, 0.2473281]
LDH	Anesthetic type	−0.7680302	0.3758261	−2.04	0.062	[−1.579953, 0.0438927]
	Time of administration	−0.0303537	0.1772381	−0.17	0.867	[−0.4132534, 0.3525461]
	Mode of administration	0.8455731	0.5484027	1.54	0.147	[−0.3391789, 2.030325]
	Duration of ischemia	−0.5446995	1.383987	−0.39	0.700	[−3.534621, 2.445223]
	Duration of reperfusion	1.217208	0.6565302	1.85	0.087	[−0.2011388, 2.635556]
CK-MB	Anesthetic type	0.0692659	0.3193602	0.22	0.834	[−0.6859009, 0.8244328]
	Time of administration	−0.924908	0.2594603	−3.56	0.009	[−1.538434,−0.3113818]
	Mode of administration	−0.6362163	0.5458382	−1.17	0.282	[−1.926918, 0.6544858]
	Duration of ischemia	4.932397	1.28456	3.84	0.006	[1.894895, 7.969899]
	Duration of reperfusion	0.4996208	0.792962	0.63	0.549	[−1.375436, 2.374678]
SOD	Anesthetic type	−0.3987254	0.3005316	−1.33	0.211	[−1.060191, 0.2627401]
	Time of administration	0.0803098	0.1672812	0.48	0.641	[−0.2878735, 0.4484932]
	Mode of administration	0.2570402	0.3759114	0.68	0.508	[−0.5703352, 1.084416]
	Duration of ischemia	−0.992319	2.538071	−0.39	0.703	[−6.578575, 4.593937]
	Duration of reperfusion	0.2271904	0.4058535	0.56	0.587	[−0.6660872, 1.120468]
MDA	Anesthetic type	−0.8061838	0.3921053	−2.06	0.070	[−1.693188, 0.0808199]
	Time of administration	0.0592337	0.3143535	0.19	0.855	[−0.6518834, 0.7703507]
	Mode of administration	1.038319	0.5369617	1.93	0.085	[−0.176373, 2.25301]
	Duration of ischemia	−1.4892	1.652382	−0.90	0.391	[−5.227148, 2.248749]
	Duration of reperfusion	0.5046542	0.5098885	0.99	0.348	[−0.6487937, 1.658102]

*IS* Infarct size, *LDH* Lactate dehydrogenase, *CK-MB* Creatine kinase MB, *SOD* Superoxide dismutase, *MDA* Malondialdehyde

### Detection of publication bias

By utilizing a funnel plot and Egger’s test, we identified significant publication bias in thirteen outcome metrics: IS (Egger’s: *t* = 7.06, *P* < 0.05), +dp/dtmax (Egger’s: *t* = 6.26, *P* < 0.05), -dp/dtmax (Egger’s: *t* = 6.34, *P* < 0.05), LVEF (Egger’s: *t* = 3.09, *P* < 0.05), LVSP (Egger’s: *t* = 8.76, *P* < 0.05), LVEDP (Egger’s: *t* = 15.44, *P* < 0.05), LDH (Egger’s: *t* = 9.27, *P* < 0.05), CK-MB (Egger’s: *t* = 6.32, *P* < 0.05), CK (Egger’s: *t* = 7.49, *P* < 0.05), SOD (Egger’s: *t* = 11.26, *P* < 0.05), apoptotic rate (Egger’s: *t* = 6.72, *P* < 0.05), TNF-α (Egger’s: *t* = 4.88, *P* < 0.05), and IL-6 (Egger’s: *t* = 26.12, *P* < 0.05). There was no significant publication bias detected for the remaining two indicator outcome metrics, as detailed in Supplementary File 1.

We employed the trim-and-fill method to analyze 13 outcome measures with publication bias. These measures included: IS (Heterogeneity: Q = 131.887, *P* < 0.01, SMD = 12.12, 95% CI = 6.43–22.84), +dp/dtmax (Heterogeneity: Q = 35.042, *P* < 0.01, SMD = 15.35, 95% CI = 5.41–43.52), -dp/dtmax (Heterogeneity: Q = 25.895, *P* = 0.004 < 0.01, SMD = 6.18, 95% CI = 3.09–12.35), LVEF (Heterogeneity: Q = 31.230, *P* = 0.001 < 0.01, SMD = 11.36, 95% CI = 3.50–36.86), LVSP (Heterogeneity: Q = 31.230, *P* = 0.001 < 0.01, SMD = 11.36, 95% CI = 3.50–36.86), LVEDP (Heterogeneity: Q = 67.817, *P* < 0.01, SMD = 12.69, 95% CI = 1.96–82.41), LDH (Heterogeneity: Q = 170.743, *P* < 0.01, SMD = 6.48, 95% CI = 2.63–15.95), CK-MB (Heterogeneity: Q = 73.086, *P* < 0.01, SMD = 20.75, 95% CI = 7.15–60.22), CK (Heterogeneity: Q = 88.185, *P* < 0.01, SMD = 89.39, 95% CI = 11.77–678.70), SOD (Heterogeneity: Q = 96.745, *P* < 0.01, SMD = 5.45, 95% CI = 2.79–10.63), apoptotic rate (Heterogeneity: Q = 19.714, *P* < 0.01, SMD = 6.47, 95% CI = 1.52–27.51), TNF-α (Heterogeneity: Q = 73.084, *P* < 0.01, SMD = 84.00, 95% CI = 11.10–635.77), and IL-6 (Heterogeneity: Q = 21.386, *P* < 0.01, SMD = 11.12, 95% CI = 2.97–41.55). Even after accounting for potentially missing studies, the results remained statistically significant, indicating that the significance of our findings is not attributable to publication bias. Detailed trim-and-fill results are provided in Supplementary File 1.

## Discussion

### Summary of evidence

This paper has synthesized and analyzed 34 studies that included a total of 505 animals. Compared with the control group, ginsenosides significantly reduced infarct size, inhibited the elevation of LVEDP, and attenuated the decreases in LVSP, +dp/dtmax, and –dp/dtmax. These findings collectively demonstrate that ginsenosides exert robust cardioprotection. In addition, ginsenosides inhibited the increases in serum levels of LDH, CK-MB, CK, MDA, TNF-α, IL-6, and IL-1β, as well as cardiomyocyte apoptosis after MIRI. Despite significant publication bias in most outcome measures, sensitivity analyses and trim-and-fill analyses indicated that our findings were robust and that the conclusions were reliable. The presence of publication bias is attributable to substantial underreporting of negative outcomes, because nearly all included studies reported positive results, which exaggerated the protective effects of ginsenosides. Additionally, almost half of the included studies had sample sizes of fewer than eight animals per group. Subgroup analyses indicated that ginsenosides demonstrated consistent therapeutic effects across animal species, ginsenoside subtypes, and routes of administration. Based on our analysis of existing literature, the promising therapeutic potential demonstrated by ginsenosides in treating MIRI cannot be overlooked.

### Mechanistic exploration

MIRI, a frequent complication of reperfusion therapy for IHD, remains an unresolved priority in cardiology. It is driven by multiple molecular mechanisms, including inflammation, oxidative stress, calcium overload, impaired energy metabolism, apoptosis, autophagy, and mitochondrial dysfunction [57, 58]. Our findings indicate that ginsenosides inhibit free radical production and inflammatory responses, reduce the size of myocardial infarction, and improve blood flow, thereby mitigating myocardial injury following reperfusion. These results are consistent with those reported by Zhu et al. [59]. In fact, the anti-MIRI benefits of ginsenosides were previously suggested. In vitro studies by Dong et al. demonstrated that ginsenosides maintained the dynamic balance of mitochondria by regulating mitofusin 2 and glutamate dehydrogenase, thereby effectively preventing hypoxia/reoxygenation-induced damage to cardiomyocytes [60]. At an earlier time, Sun and colleagues found that pretreatment with ginsenosides increased the viability of rat cardiomyocytes, reduced LDH release, caspase-3 activity, and apoptosis, and that this protective effect was closely related to the AKT/MAPK signaling pathway [61]. All aforementioned evidence indicates that ginsenosides are potential anti-MIRI agents, and our results confirm this.

With advances in medical research, the anti-MIRI effects of ginsenosides have garnered increasing attention. The MIRI process entails dysfunction of cation pumps, calcium overload, and excessive reactive oxygen species, which impair energy metabolism and up-regulate autophagy [62, 63]. Research has shown that ginsenosides inhibit the opening of the mitochondrial permeability transition pore, restore mitochondrial membrane potential, and upregulate phosphorylated protein kinase B and phosphorylated glycogen synthase kinase-3β, thereby correcting energy metabolism disorders in cardiomyocytes [64]. Ginsenosides also regulate the expression of proteins in the tricarboxylic acid cycle, including the L-lactate dehydrogenase B chain, the pyruvate dehydrogenase complex, and the aldose reductase, thereby enhancing energy metabolism in ischemic rat hearts [65]. In addition, ginsenosides activate an autophagy-mediated survival response that prevents cardiomyocyte apoptosis. Beclin 1 and Bcl-2, key regulators of autophagy, initiate autophagy during starvation; however, ginsenosides weaken their interaction in H9c2 cells under starvation, thereby reducing autophagy and preventing apoptosis [66]. Besides these two pathways, ginsenosides enhance adenosine triphosphatase activity in cardiomyocytes and regulate cytoplasmic–endoplasmic-reticulum ion homeostasis, thereby attenuating myocardial damage induced by calcium overload [67]. In summary, primary pharmacologically active constituents of ginseng, ginsenosides exhibit significant therapeutic potential.

### Strengths and prospects

Ginseng has been extensively studied as a potential treatment for MIRI because of its multi-component, multi-pathway, and multi-target mode of action [68]. Studies have demonstrated that ginsenosides, the principal bioactive constituents of ginseng, directly interact with diverse immune cells and thereby confer therapeutic effects against MIRI. Furthermore, ginsenosides exhibit high bioactivity, are rapidly absorbed and utilized, and cause minimal adverse effects [69, 70]. The anti-MIRI properties of ginsenosides were clearly evident from our subgroup analysis. However, certain subgroups were represented by only a single study, which limits their informative value. More importantly, we found that administering ginsenosides before ischemia-reperfusion was more protective than administering them after. Additionally, when comparing different ginsenoside components, ginsenoside Rg exhibited superior efficacy in reducing LDH levels, whereas ginsenoside Rh provided better cost-effectiveness in lowering CK-MB levels. The core findings of this study support previous research indicating that ginsenosides exhibit promising therapeutic potential for MIRI [37, 71]. This consistency across studies and periods underscores the maturity of the field. Our contribution lies in enlarging the sample size with additional studies, thereby improving the precision of effect estimates. Additionally, we applied the GRADE framework to grade confidence in our conclusions and to transparently acknowledge the suboptimal quality of evidence for some outcomes.

### Translational implications

In the modern drug development process, systematic reviews and meta-analyses of animal studies represent a crucial step toward translating preclinical data into clinical applications. The literature included herein is highly consistent with clinical MIRI cases, both in terms of disease model construction and the simulation of pathophysiological processes. Nevertheless, animal models inherently differ from human pathophysiology; therefore, verifying the efficacy of ginsenosides solely in animal models is insufficient. Phase I clinical trials are warranted to assess their safety profiles and validate our findings. Moreover, the current understanding of the mechanisms underlying ginsenoside action focuses primarily on anti-inflammatory, anti-apoptotic, and antioxidative stress effects, whereas calcium overload, energy metabolism disorders, ferroptosis, and autophagy remain understudied. We therefore recommend that future research prioritize these underinvestigated pathways as potential therapeutic targets.

### Limitations

This study is based on extensive preclinical evidence that illustrates the significant effects of ginsenosides against MIRI, and confirms their efficacy. Nevertheless, several limitations of the present work should be recognized. First, most included studies exhibited low or unclear methodological quality, with inadequate reporting of blinding and risk of bias; consequently, some indicators received low-quality ratings that may weaken the persuasiveness of our conclusions. Second, substantial heterogeneity affected most outcome indicators; despite our efforts to identify its sources, limited literature precluded comprehensive analysis for some outcomes. Third, most corresponding authors are affiliated with Chinese institutions, and this geographical concentration may increase heterogeneity. Finally, the use of SMD and pooling across different doses may obscure dose–response relationships; thus, caution is warranted when interpreting these results. Additionally, MIRI patients often present with comorbidities such as hypertension and diabetes; however, this factor was rarely accounted for in the included studies. Therefore, future research should develop models that incorporate these pathological characteristics to evaluate whether ginsenoside-based combination therapy enhances cardioprotective efficacy.

## Conclusion

This study systematically evaluated the preventive and therapeutic effects of ginsenosides against MIRI. Preliminary findings indicate that ginsenosides reduce myocardial infarct size and improve cardiac function. Moreover, ginsenosides exhibit potent anti-inflammatory, anti-apoptotic, and antioxidant properties. Despite its limitations, the outlook for ginsenosides as therapeutic agents against MIRI remains promising. Thus, ginsenosides may offer novel preventive and therapeutic strategies for patients with MIRI, warranting further investigation and development.

## Supplementary Information

Supplementary Material 1. Supplementary Figure 1: Sensitivity analysis of myocardial infarction size.

Supplementary Material 2. Supplementary Figure 2: Sensitivity analysis of the effect of ginsenosides on cardiac function. (A) +dp/dtmax, (B) -dp/dtmax, (C) LVEF, (D) LVSP, (E) LVEDP.

Supplementary Material 3. Supplementary Figure 3: Sensitivity analysis of the effect of ginsenosides on myocardial injury. (A) LDH, (B) CK-MB, (C) CK.

Supplementary Material 4. Supplementary Figure 4: Sensitivity analysis of ginsenosides on the effects of apoptosis and oxidative stress. (A) Apoptosis rate, (B) SOD, (C) MDA.

Supplementary Material 5. Supplementary Figure 5: Sensitivity analysis of the effect of ginsenosides on myocardial inflammation. (A) IL-1β, (B) IL-6, (C) TNF-α.

Supplementary Material 6. Supplementary File 1: Results of funnel plots, Egger’s test, and trim-and-fill analyses.

## Data Availability

The raw data shown in this study have been provided in the article or supplementary material. Additional data can be obtained by contacting the corresponding author upon reasonable request.

## Abbreviations

IHD: Ischemic heart disease
MIRI: Myocardial ischemia-reperfusion injury
I^2^: I squared
CI: Confidence interval
SMD: Standardized mean difference
WMD: Weighted mean difference
LVSP: Left ventricular systolic pressure
LVEDP: Left ventricular end-diastolic pressure
+dp/dtmax: Maximum rate of left ventricular pressure rise
-dp/dtmax: Maximum rate of left ventricular pressure decrease
LVEF: Left ventricular ejection fraction
SOD: Superoxide dismutase
MDA: Malondialdehyde
CK-MB: Creatine kinase MB
CK: Creatine kinase
IL-1β: Interleukin-1β
IL-6: Interleukin-6
TNF-α: Tumor necrosis factor-alpha
PROSPERO: International prospective register of systematic reviews
PICOS: Population, intervention, comparison, outcomes, study design
SYRCLE: Systematic Review Center for Laboratory animal Experimentation
GRADE: Grading of Recommendations, Assessment, Development, and Evaluations
