# Supplementary figures and images for: Preventive and therapeutic effects of ginsenosides on myocardial ischemia-reperfusion injury in animal models: a systematic review and meta-analysis
Source: BMC Cardiovasc Disord. 2026 Jan 14;26:132. doi: 10.1186/s12872-026-05503-7 (PMC12888158; doi:10.1186/s12872-026-05503-7)

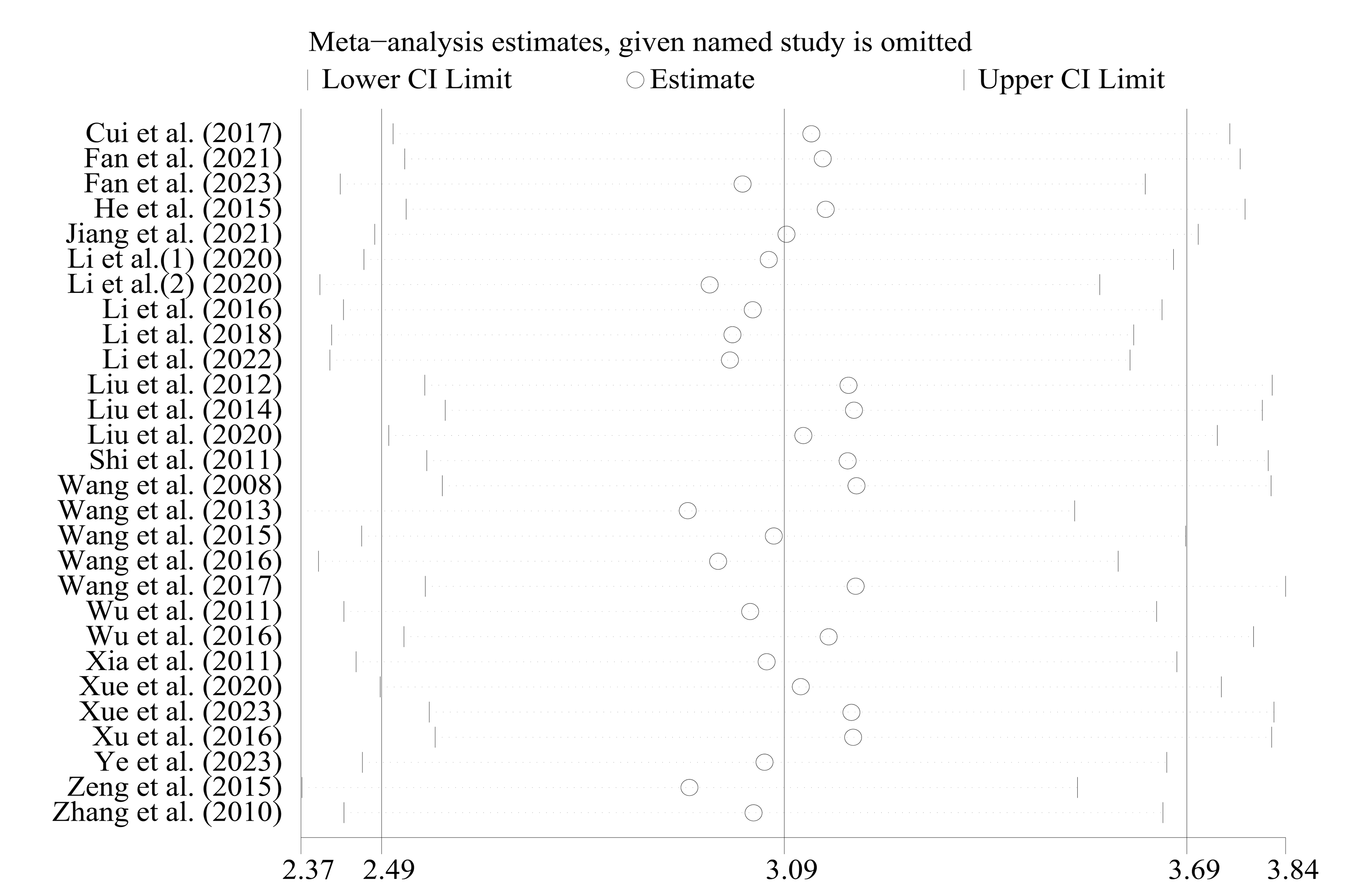

Supplement: Supplementary file 1 — Supplementary Material 1. Supplementary Figure 1: Sensitivity analysis of myocardial infarction size. [file 12872_2026_5503_MOESM1_ESM.tif]

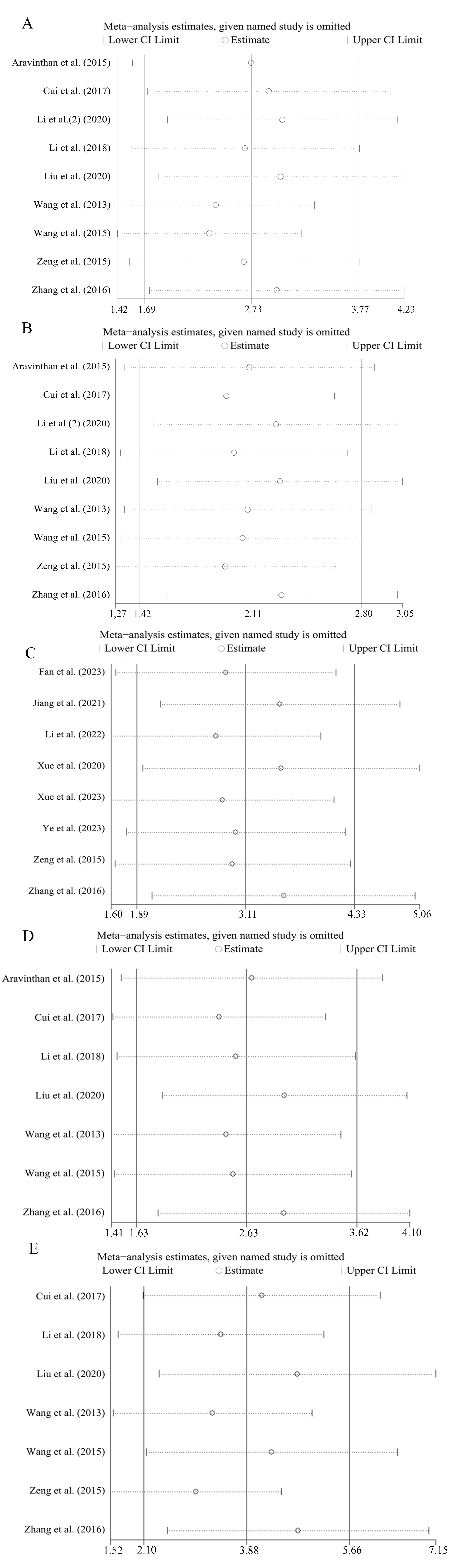

Supplement: Supplementary file 2 — Supplementary Material 2. Supplementary Figure 2: Sensitivity analysis of the effect of ginsenosides on cardiac function. (A) +dp/dtmax, (B) -dp/dtmax, (C) LVEF, (D) LVSP, (E) LVEDP. [file 12872_2026_5503_MOESM2_ESM.tif]

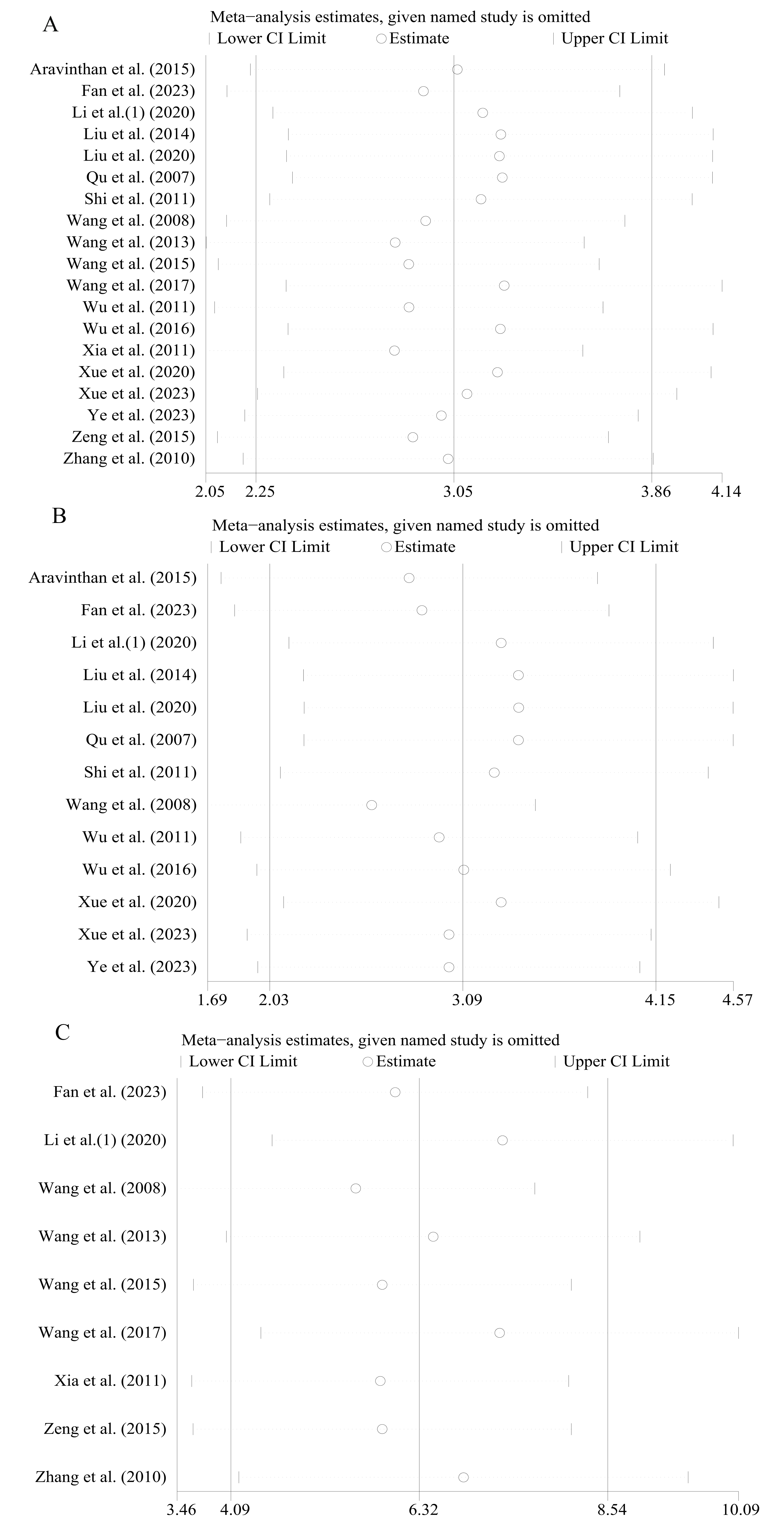

Supplement: Supplementary file 3 — Supplementary Material 3. Supplementary Figure 3: Sensitivity analysis of the effect of ginsenosides on myocardial injury. (A) LDH, (B) CK-MB, (C) CK. [file 12872_2026_5503_MOESM3_ESM.tif]

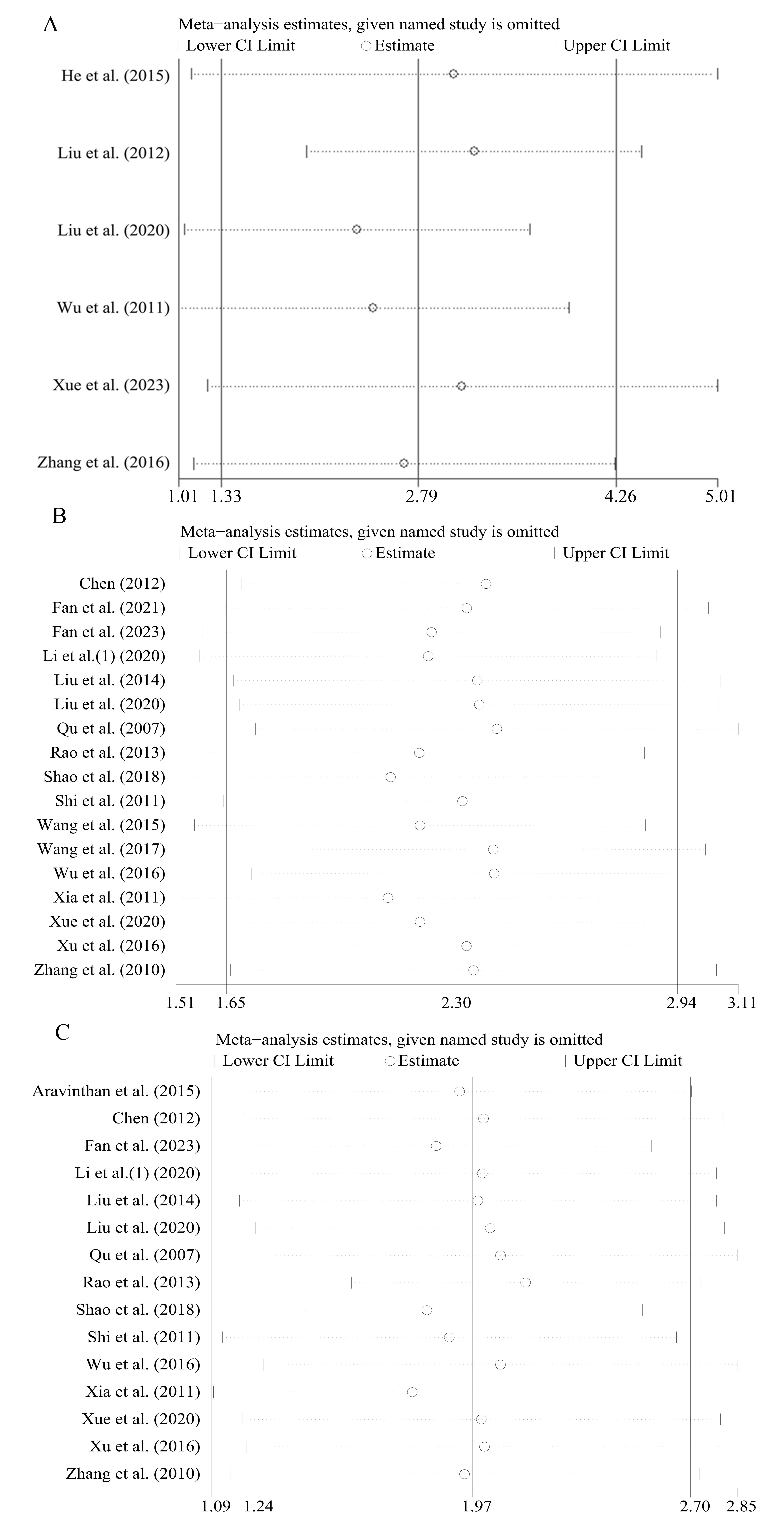

Supplement: Supplementary file 4 — Supplementary Material 4. Supplementary Figure 4: Sensitivity analysis of ginsenosides on the effects of apoptosis and oxidative stress. (A) Apoptosis rate, (B) SOD, (C) MDA. [file 12872_2026_5503_MOESM4_ESM.tif]

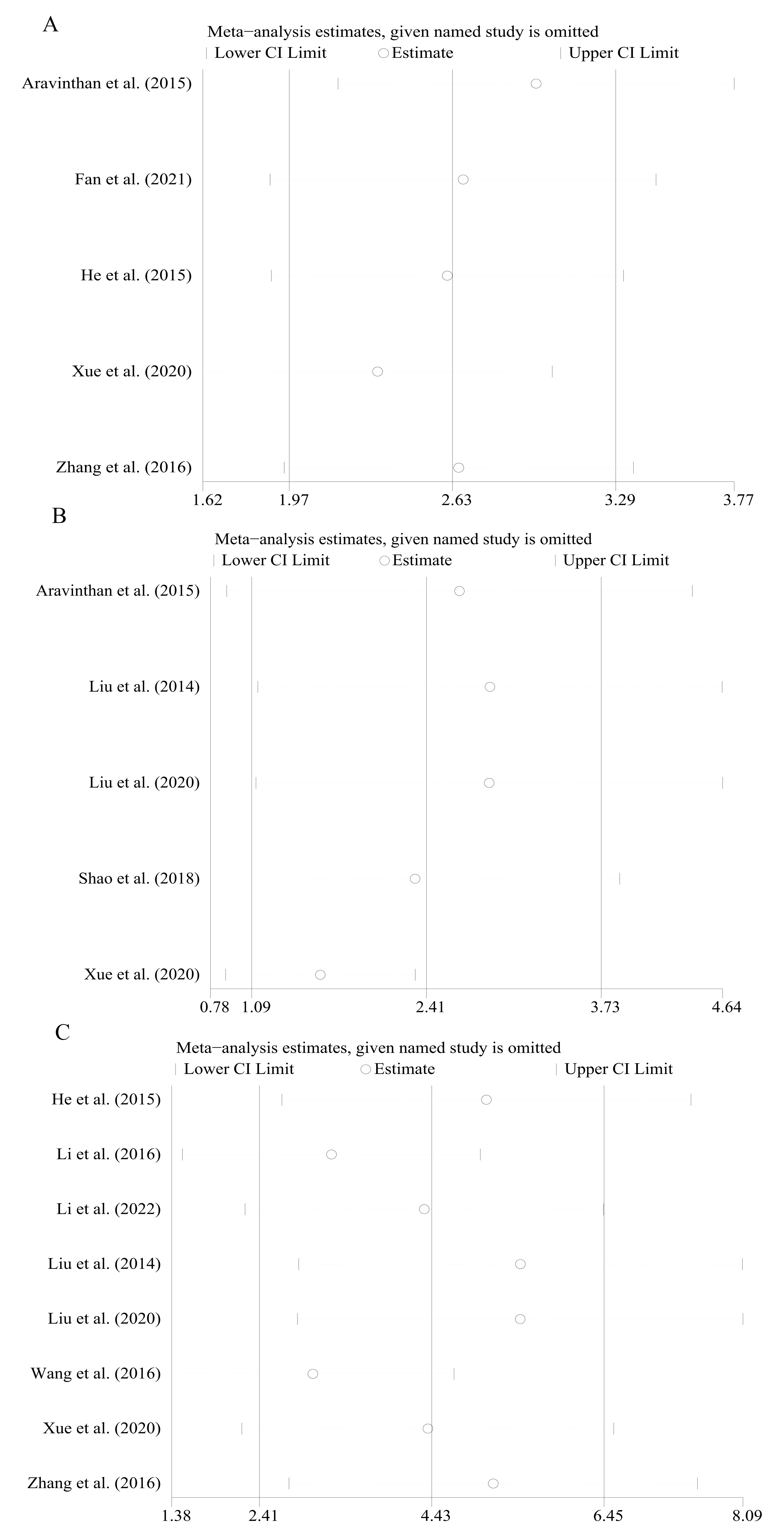

Supplement: Supplementary file 5 — Supplementary Material 5. Supplementary Figure 5: Sensitivity analysis of the effect of ginsenosides on myocardial inflammation. (A) IL-1β, (B) IL-6, (C) TNF-α. [file 12872_2026_5503_MOESM5_ESM.tif]
