# Supplementary material for: Preventive and therapeutic effects of ginsenosides on myocardial ischemia-reperfusion injury in animal models: a systematic review and meta-analysis
Source: BMC Cardiovasc Disord. 2026 Jan 14;26:132. doi: 10.1186/s12872-026-05503-7 (PMC12888158; doi:10.1186/s12872-026-05503-7)
Supplement: Supplementary file 6 — Supplementary Material 6. Supplementary File 1: Results of funnel plots, Egger’s test, and trim-and-fill analyses. [file 12872_2026_5503_MOESM6_ESM.docx]

Supplementary File 1: Funnel Plots and Egger’s Test Results


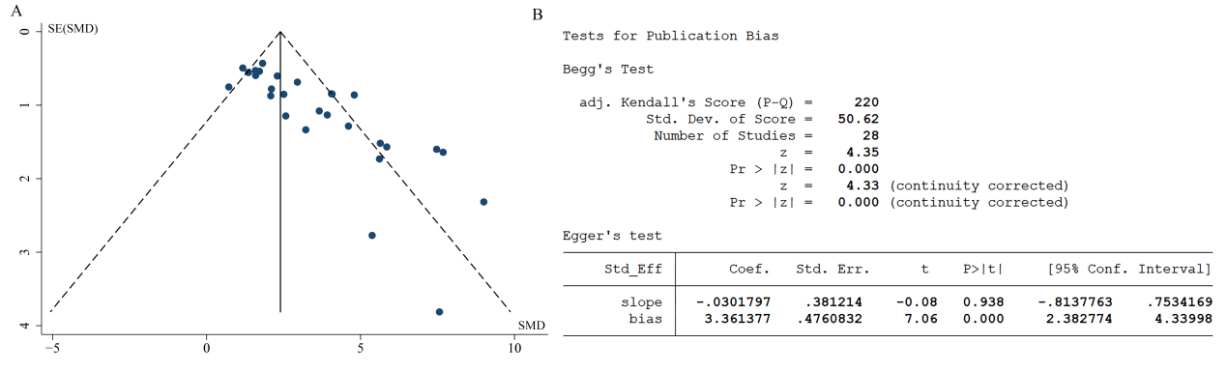


(A) Funnel plot and (B) Egger's and Begg's test values of myocardial infarction size.


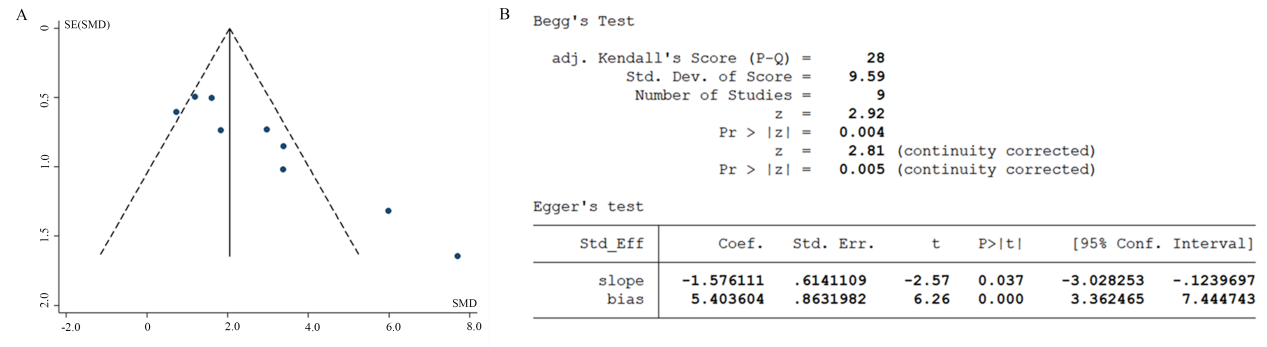


(A) Funnel plot and (B) Egger's and Begg's test values of +dp/dtmax.


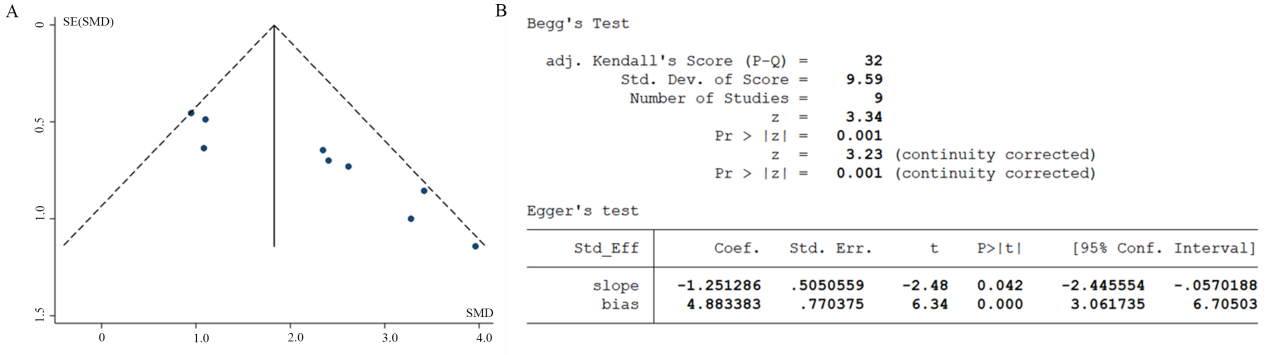


(A) Funnel plot and (B) Egger's and Begg's test values of -dp/dtmax.


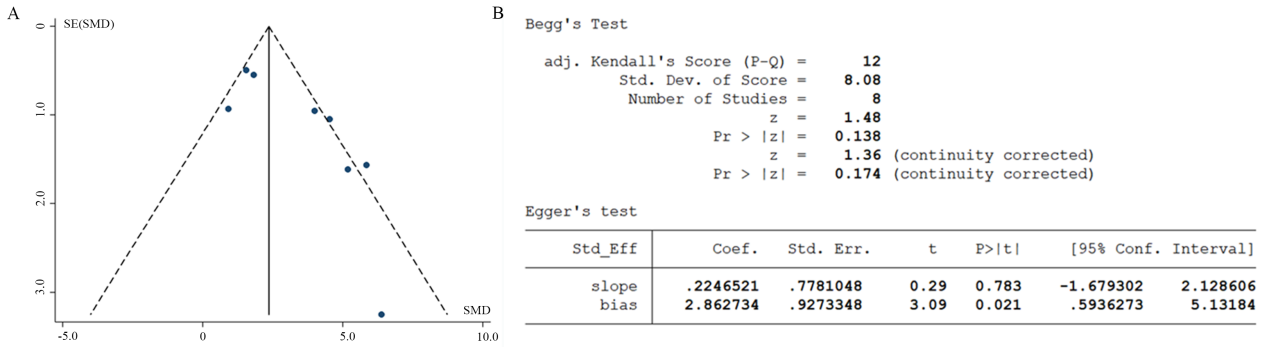


(A) Funnel plot and (B) Egger's and Begg's test values of LVEF.


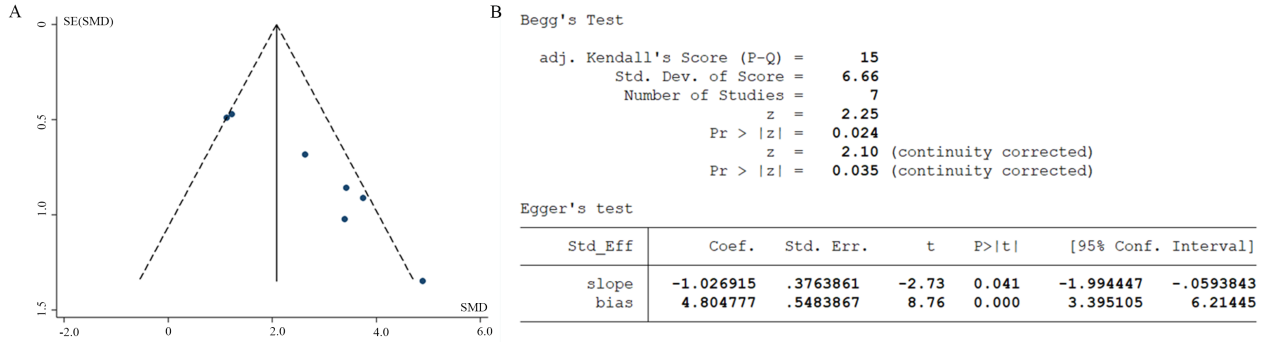


(A) Funnel plot and (B) Egger's and Begg's test values of LVSP.


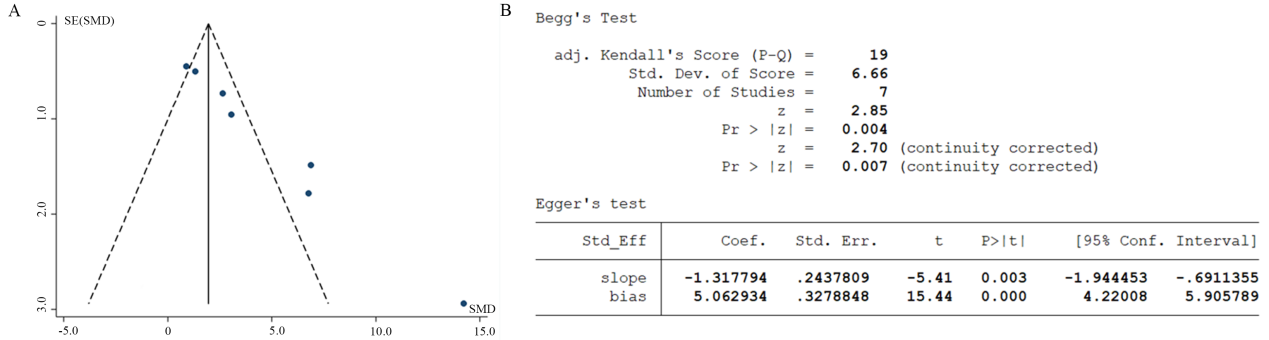


(A) Funnel plot and (B) Egger's and Begg's test values of LVEDP.


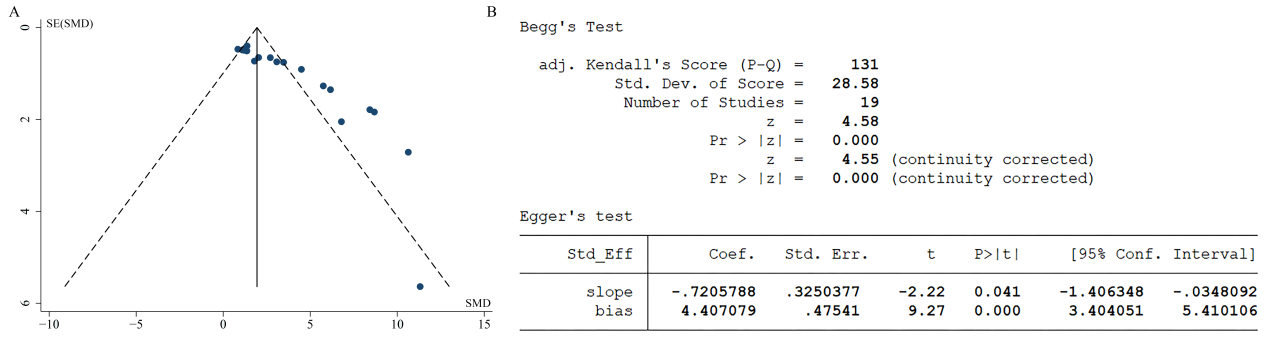


(A) Funnel plot and (B) Egger's and Begg's test values of LDH.


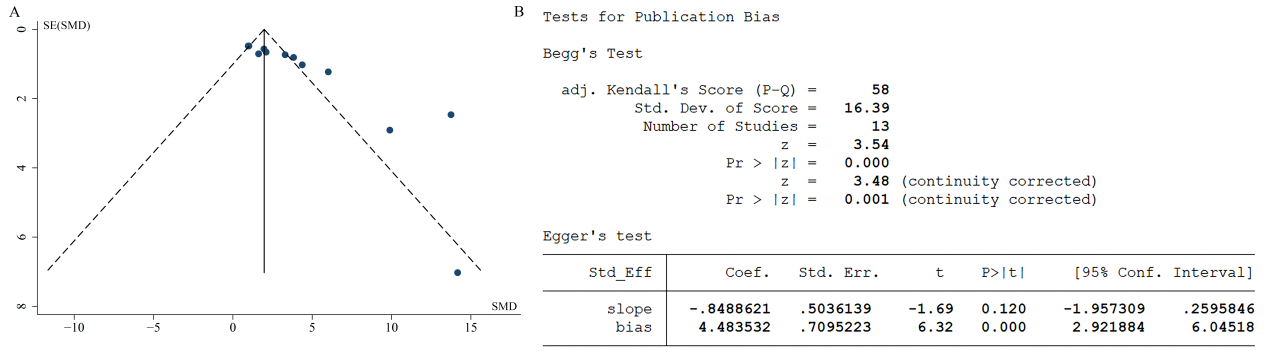


(A) Funnel plot and (B) Egger's and Begg's test values of CK-MB.


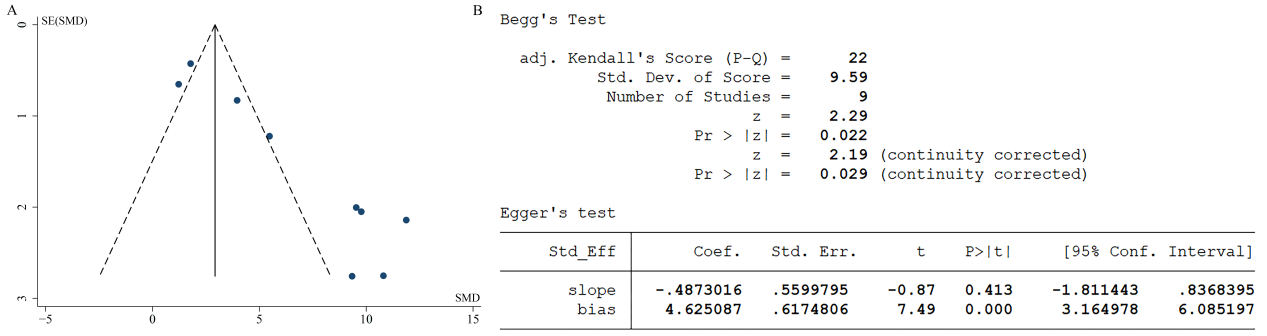


(A) Funnel plot and (B) Egger's and Begg's test values of CK.


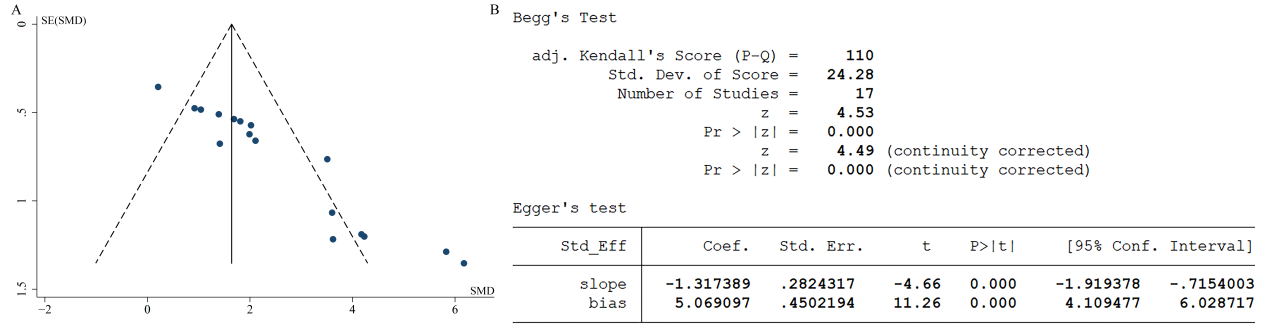


(A) Funnel plot and (B) Egger's and Begg's test values of SOD.


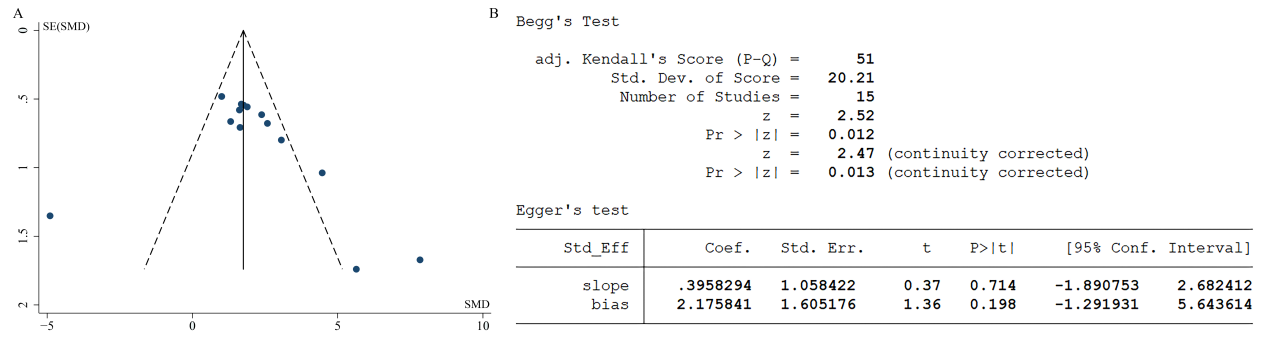


(A) Funnel plot and (B) Egger's and Begg's test values of MDA.


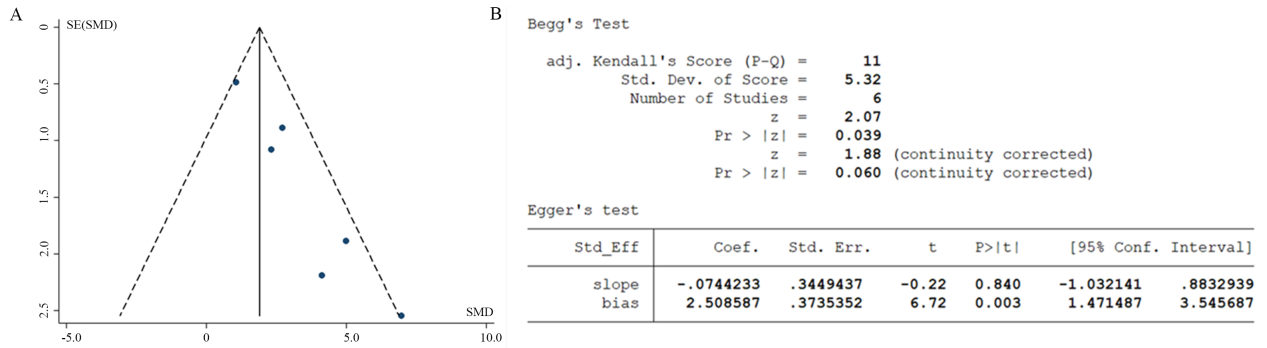


(A) Funnel plot and (B) Egger's and Begg's test values of apoptotic rate.


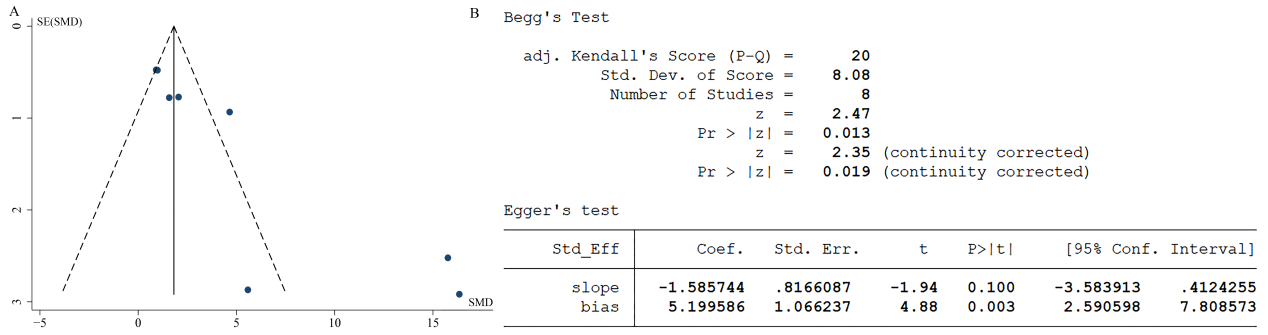


(A) Funnel plot and (B) Egger's and Begg's test values of TNF-α.


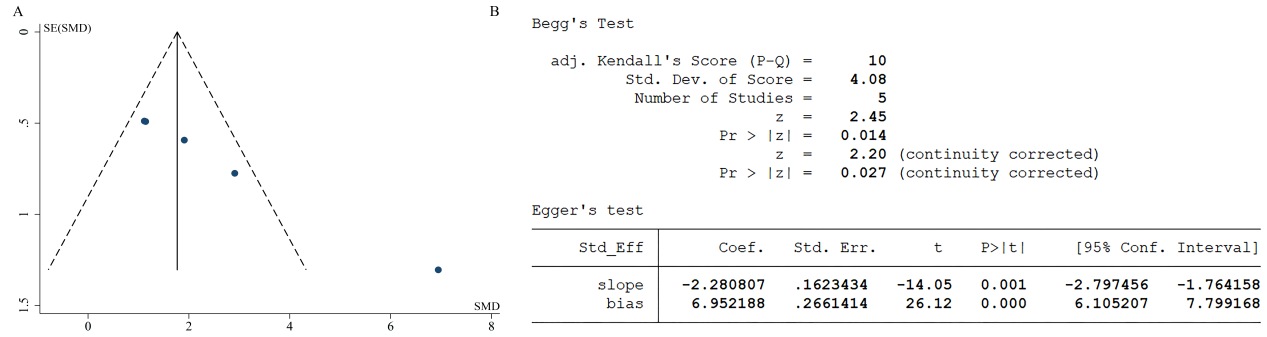


(A) Funnel plot and (B) Egger's and Begg's test values of IL-6.


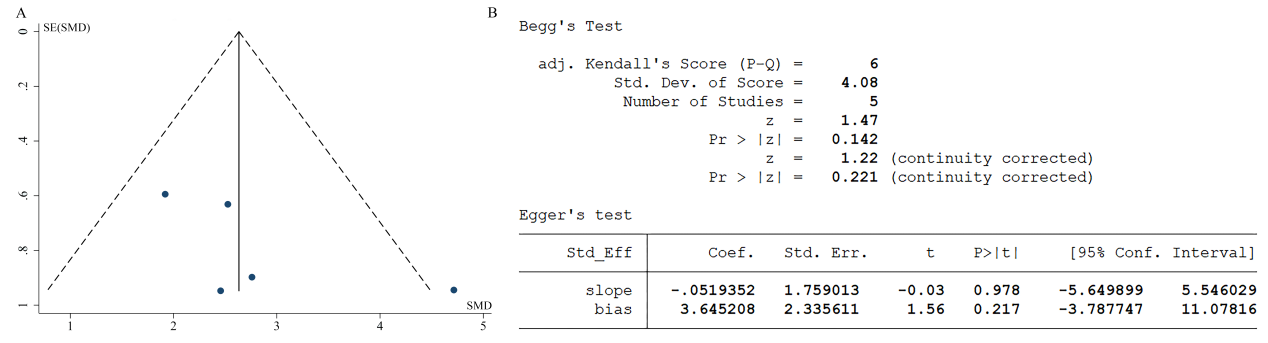


1. Funnel plot and (B) Egger's and Begg's test values of IL-1β.

Supplementary File 1: Trim and Fill Analysis Results


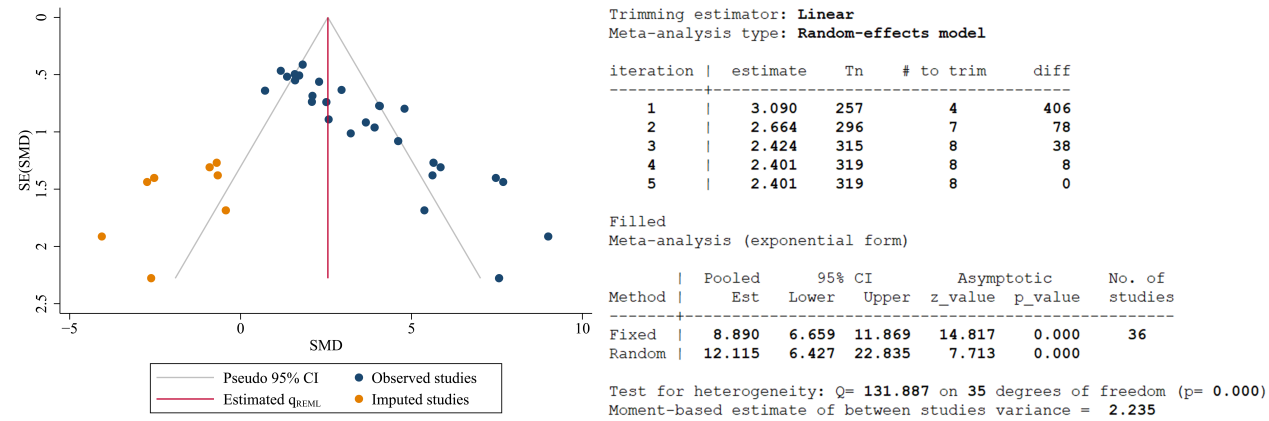


1. Results of the trim-and-fill analysis of myocardial infarction size.


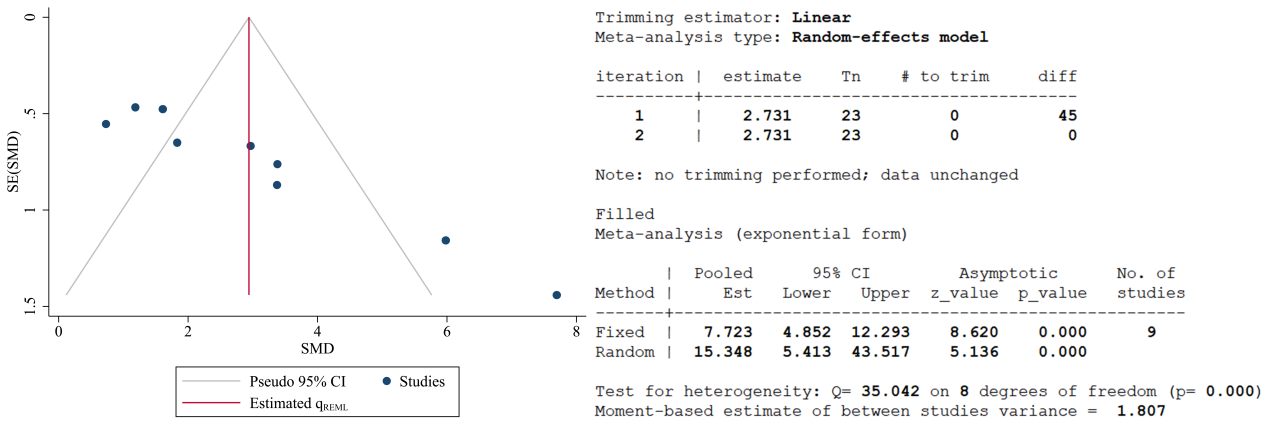


1. Results of the trim-and-fill analysis of +dp/dtmax.


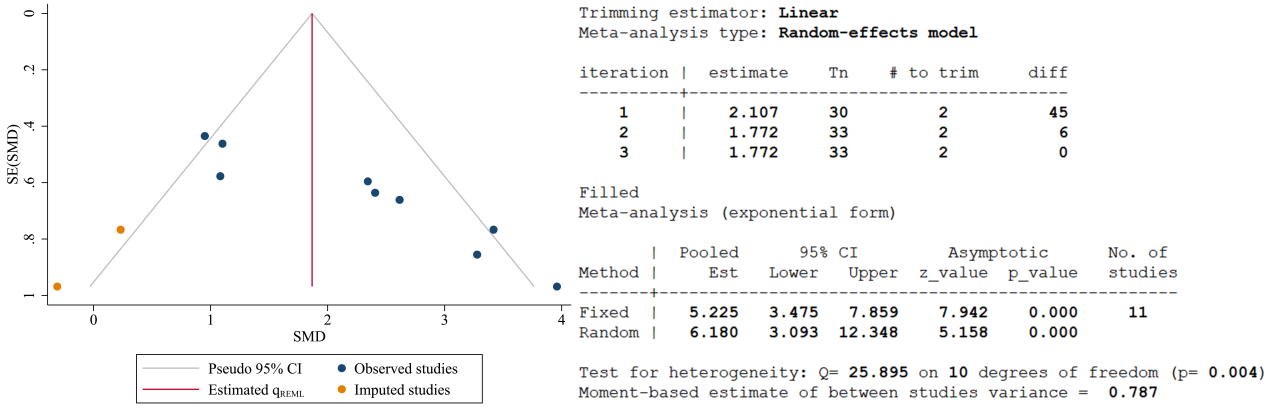


1. Results of the trim-and-fill analysis of -dp/dtmax.


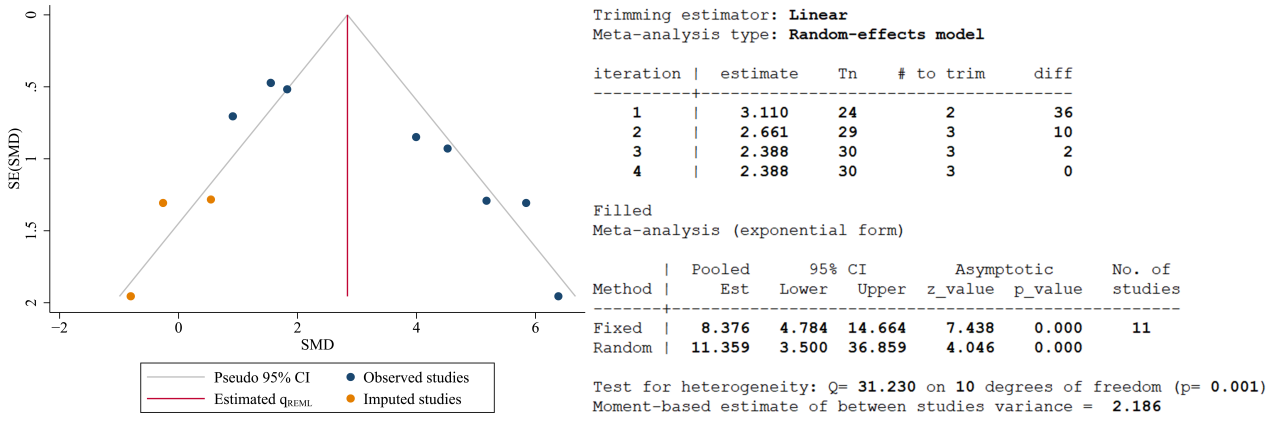


1. Results of the trim-and-fill analysis of LVEF.


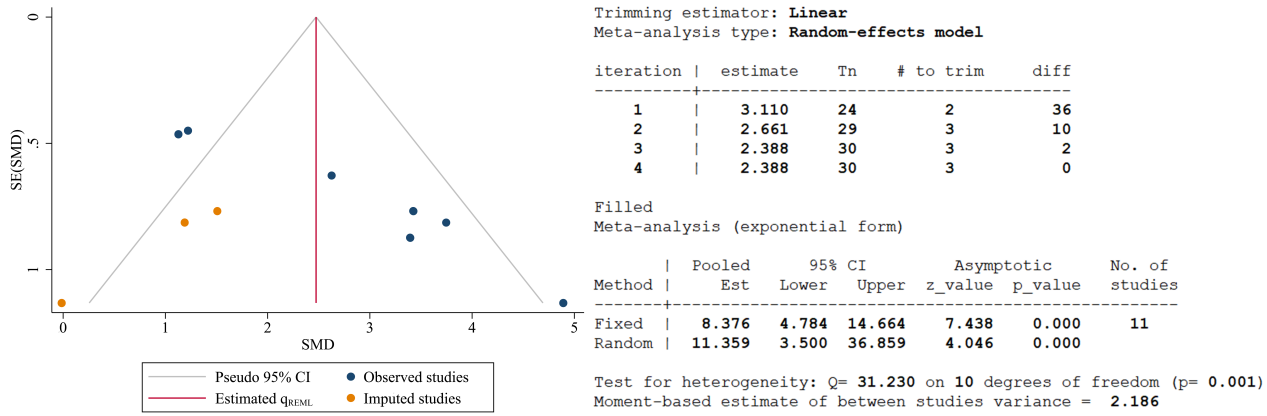


1. Results of the trim-and-fill analysis of LVSP.


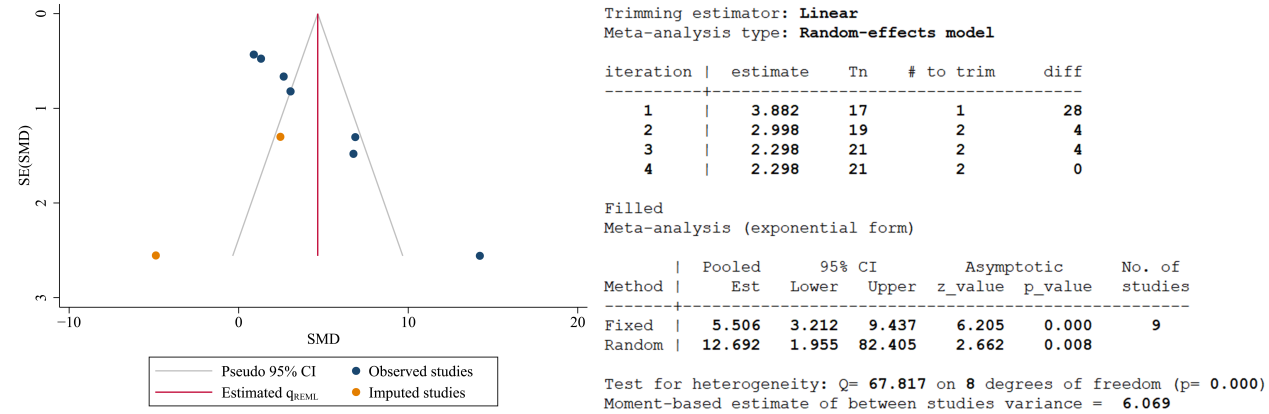


1. Results of the trim-and-fill analysis of LVEDP.


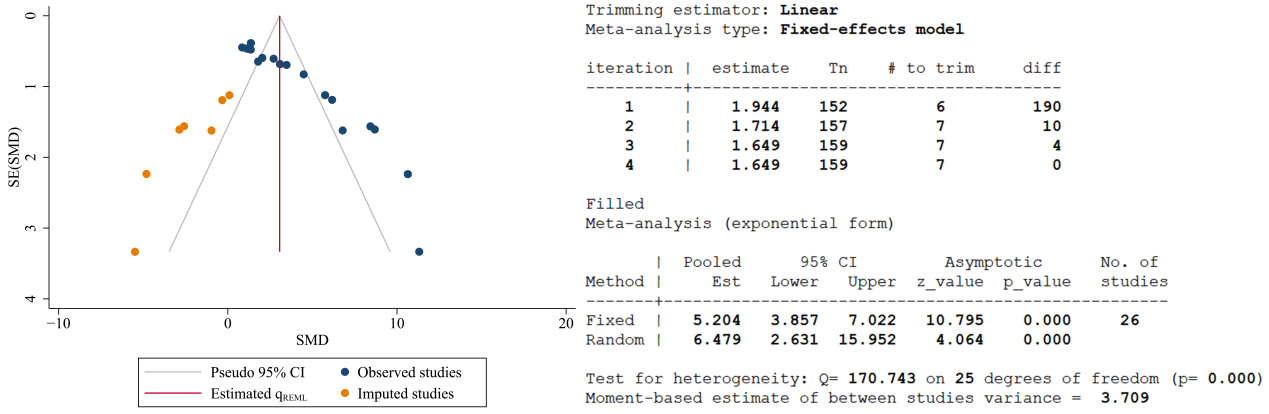


1. Results of the trim-and-fill analysis of LDH.


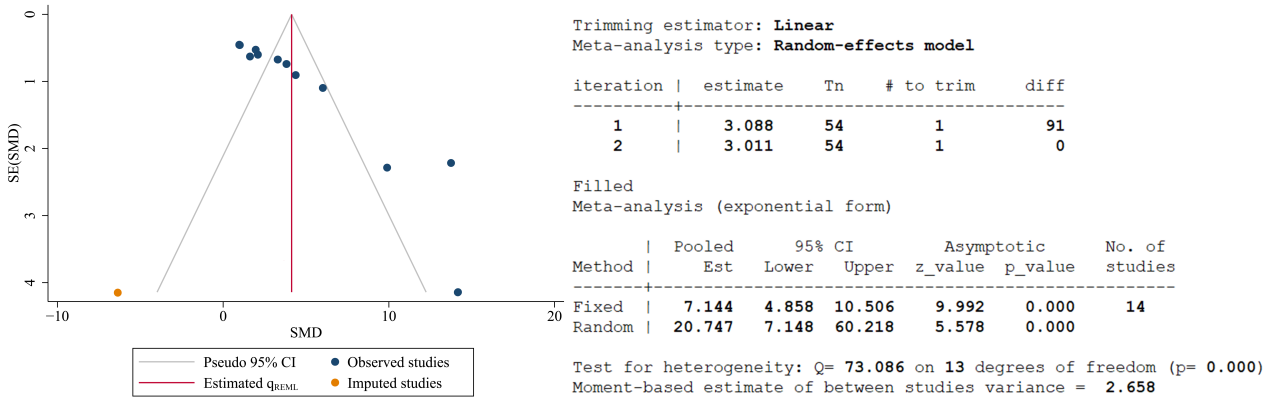


1. Results of the trim-and-fill analysis of CK-MB.


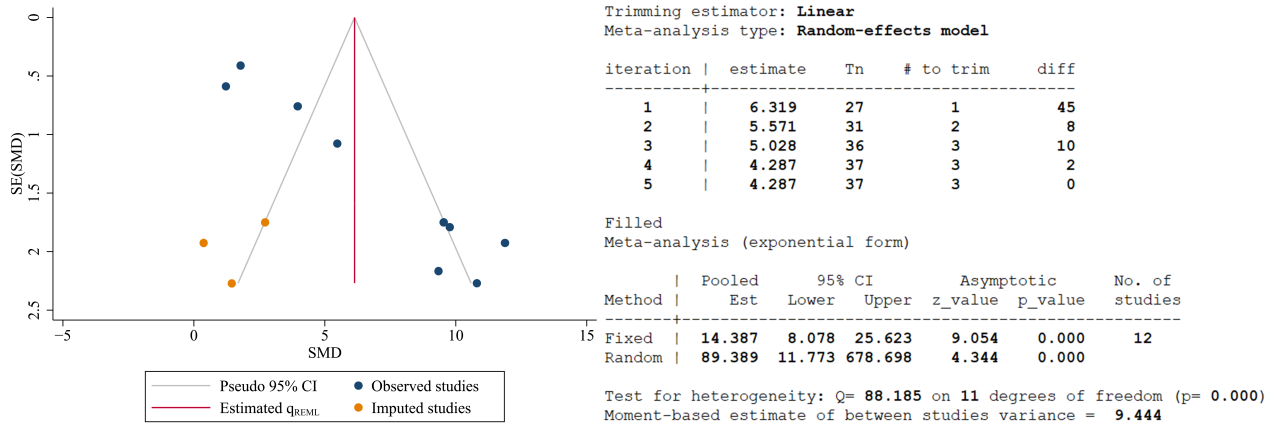


1. Results of the trim-and-fill analysis of CK.


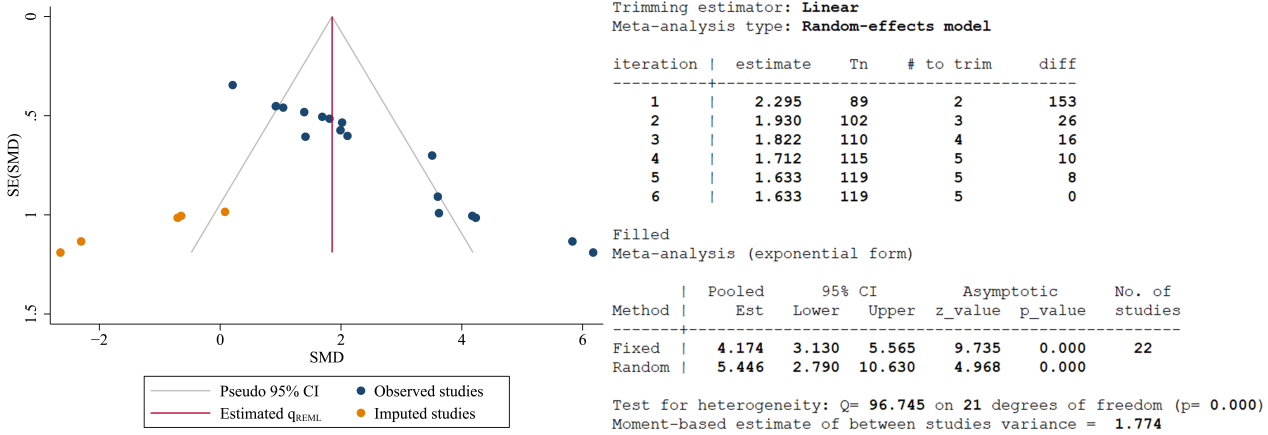


1. Results of the trim-and-fill analysis of SOD.


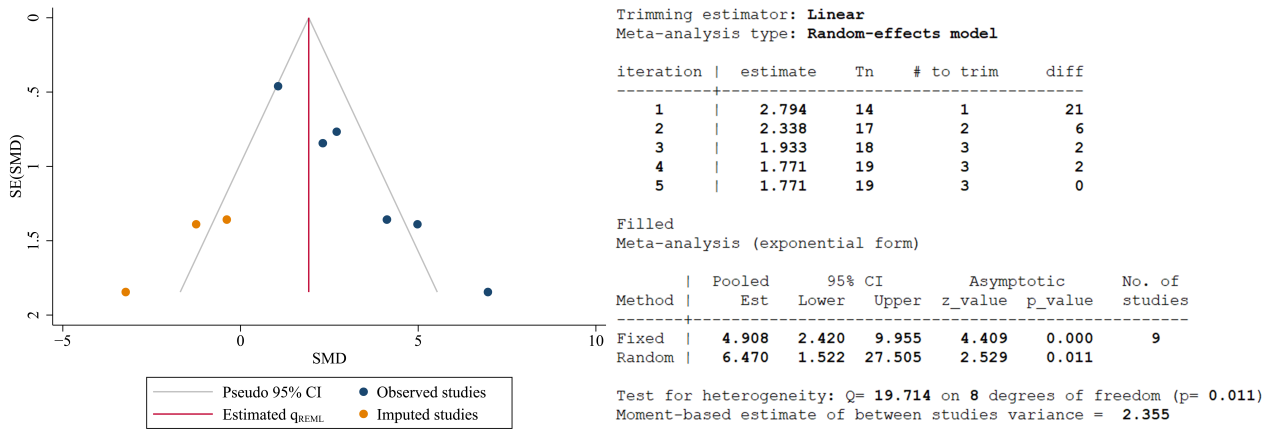


1. Results of the trim-and-fill analysis of apoptotic rate.


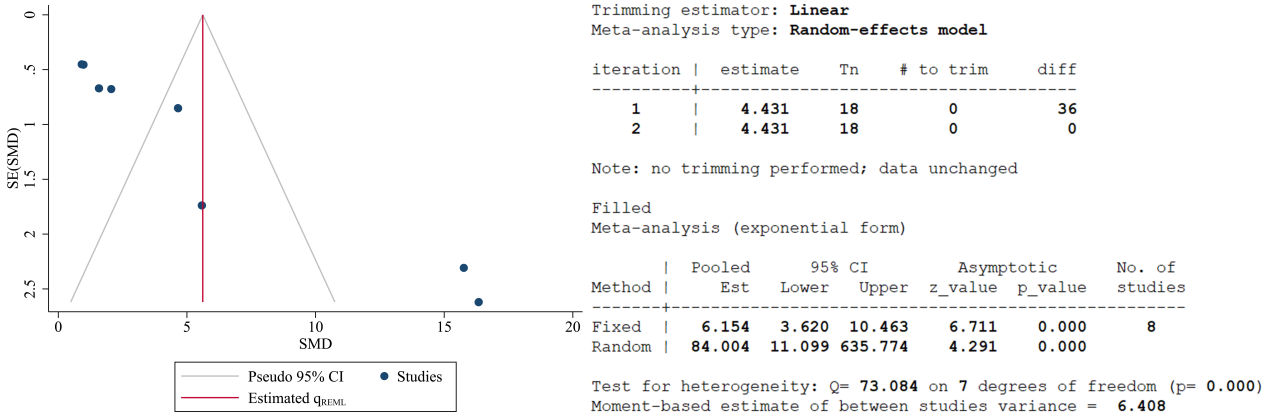


1. Results of the trim-and-fill analysis of TNF-α.


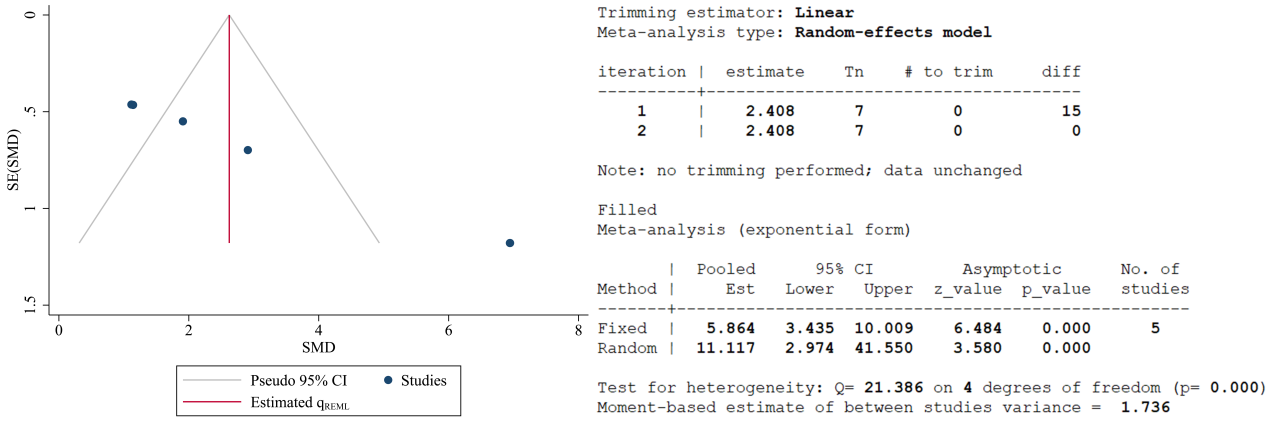


1. Results of the trim-and-fill analysis of IL-6.
